# Assembly-induced spin transfer and distance-dependent spin coupling in atomically precise AgCu nanoclusters

**DOI:** 10.1038/s41467-022-33651-9

**Published:** 2022-10-08

**Authors:** Nan Xia, Jianpei Xing, Di Peng, Shiyu Ji, Jun Zha, Nan Yan, Yan Su, Xue Jiang, Zhi Zeng, Jijun Zhao, Zhikun Wu

**Affiliations:** 1 Key Laboratory of Materials Physics, Anhui Key Laboratory of Nanomaterials and Nanotechnology, Institute of Solid State Physics, HFIPS, Chinese Academy of Sciences, Hefei, 230031 P. R. China; 2 Institute of Physical Science and Information Technology, Anhui University, Hefei, 230601 P. R. China; 3 Key Laboratory of Material Modification by Laser, Ion and Electron Beams (Dalian University of Technology), Ministry of Education, Dalian, 116024 P. R. China; 4 University of Science and Technology of China, Hefei, 230601 P. R. China

**Keywords:** Nanoparticles, Structural properties, Magnetic materials

## Abstract

Nanoparticle assembly paves the way for unanticipated properties and applications from the nanoscale to the macroscopic world. However, the study of such material systems is greatly inhibited due to the obscure compositions and structures of nanoparticles (especially the surface structures). The assembly of atomically precise nanoparticles is challenging, and such an assembly of nanoparticles with metal core sizes strictly larger than 1 nm has not been achieved yet. Here, we introduced an on-site synthesis-and-assembly strategy, and successfully obtained a straight-chain assembly structure consisting of Ag_77_Cu_22_(CHT)_48_ (CHT: cyclohexanethiolate) nanoparticles with two nanoparticles separated by one S atom, as revealed by mass spectrometry and single crystal X-ray crystallography. Although Ag_77_Cu_22_(CHT)_48_ bears one unpaired shell-closing electron, the magnetic moment is found to be mainly localized at the S linker with magnetic isotropy, and the sulfur radicals were experimentally verified and found to be unstable after disassembly, demonstrating assembly-induced spin transfer. Besides, spin nanoparticles are found to couple and lose their paramagnetism at sufficiently short inter-nanoparticle distance, namely, the spin coupling depends on the inter-nanoparticle distance. However, it is not found that the spin coupling leads to the nanoparticle growth.

## Abstract (web summary)

The assembly of atomically precise clusters into ordered superstructures enables new functional material designs. Here, the authors propose a strategy for linear arrangements of AgCu clusters and explore the consequent transfer and coupling of magnetic spins.

## Introduction

Metal nanoparticles can be regarded as an assembly of metal atoms and surface-protecting ligands^1,2^. The further assembly of nanoparticles enhances or even enriches the properties of sole metal nanoparticles and constitutes a novel type of promising material that is significant not only for fundamental research but also for practical applications^2–17^. Due to numerous atoms and complex structures as well as instability, it is generally difficult to assemble metal nanoparticles in a controlled and atomically precise way^18–20^. Even the controlled and atomically precise synthesis of metal nanoparticles has long been a target for the scientific community, and this target has only come into becoming a reality recently after many trials^21^. Atomically precise nanoparticle assemblies provide excellent opportunities for structure (composition)-property correlation and rational tailoring of their performances, thus attracting extensive interest in recent years^22–32^. Unfortunately, despite the successful assembly of small metal nanoclusters (NCs) with sizes less than 1 nm^33–38^, the assembly of atomically precise metal nanoparticles with metal core sizes strictly larger than 1 nm has not yet been reported due to the increasing challenge in synthesis and characterization (assembly without linkers in crystalline phases is not considered herein). To resolve these challenging issues, the linker is one critical factor for consideration. A relatively short (simple) linker is preferred in simplifying assembly and facilitating characterization, and the most ideal linker in this context is a single atom (including charged and uncharged atoms). Cations have been widely applied in previous assemblies;^11,17^ however, anions such as S^2–^ and N^3–^ have rarely been employed as linkers, especially for the assembly of particles. S^2–^ has a good affinity to metal atoms^39,40^ and it can be in-situ produced during the synthesis of thiolated metal nanoclusters^41^. It is possible that the on-site produced sulfur anion acts as a linker to stick the concurrently produced nanoparticles during the synthesis of nanoparticles (see Supplementary Fig. 1). Another advantage of S^2–^ as a linker is that the good affinity of S^2–^ towards metal atoms can enhance or even give rise to new properties for the nanoparticles. Fortunately, for the first time, we successfully assembled nanoparticles into straight chains by using S^2–^ as a linker, resolved the atomic structure by using mass spectrometry and single crystal X-ray crystallography (SCXC), and, more importantly, revealed interesting spin transfer and coupling phenomena, which will be discussed below.

## Results

### Synthesis and structure characterization

Silver salt was employed as the major metal precursor due to its accessibility, and a second metal, copper salt, was added to strengthen the stability and enrich the properties of the metal nanoparticles. Cyclohexanethiol (CHTH) was employed as a protecting ligand due to its relative flexibility, which is beneficial for S^2–^ access to the metal surface. A kinetical control and thermodynamic selection strategy was applied to synthesize atomically precise bimetal nanoparticles^42^. Briefly, the synthesis and assembly processes are shown below: a freshly prepared solution of NaBH_4_ and PPh_4_Br was added dropwise to a dichloromethane suspension containing AgCHT and CuCl in the presence of triethylamine. After stirring for 6 h at 0 °C, a dark brown solution was obtained, which was rotary evaporated to give a dark solid. This solid was washed with methanol and then dissolved in a mixed solvent of dichloromethane/hexane for crystallization (assembly) (see Methods for details).

The UV/Vis/NIR spectra for the as-prepared materials and the crystals both show a prominent peak at 655 nm and a shoulder peak at 410 nm (Supplementary Fig. 2). Electrospray ionization mass spectrometry (ESI-MS) was employed to determine the exact formula of the components. The full range of the ESI-MS spectrum (acquired in positive ion mode) is shown in Supplementary Fig. 3a. A series of peaks were observed at approximately 4900 and 7400 Da bearing 3+ and 2+ charges, respectively. Detailed assignments are revealed in Supplementary Fig. 3b, and the isotope patterns for the parent cluster and its fragments match well with the calculated patterns (see Supplementary Figs. 4 and 5). The peak of intact cluster ion was found at m/z = 7617.7 Da, corresponding to [Ag_77_Cu_22_(CHT)_48_]^2+^, and the peak of 3+ charged cluster ion with one missing CHT ligand was located at m/z = 5040.6 Da. The other peaks can be rationally assigned to the fragments, among which two peaks are assigned to [Ag_76_Cu_21_(CHT)_47_ + S]^3+^ and [Ag_76_Cu_21_(CHT)_48_ + S]^2+^ (Supplementary Fig. 6), indicating the possible existence of surface S^2–^. The replacement of the reducing reagent NaBH_4_ with NaBD_4_ in the synthesis does not lead to the notable change of m/z in the mass spectrum of Ag_77_Cu_22_ (Supplementary Fig. 7), excluding the existence of hydrides in the metallic core. SCXC (vide infra) was used to determine the nanoparticle composition [Ag_77_Cu_22_(CHT)_48_]^2+^, which was also supported by additional EDX measurements (Ag/Cu/S atomic ratio 1: 0.285: 0.637, in good agreement with the expected ratio 1: 0.286: 0.623, see Supplementary Fig. 8). Below, we will focus on the structure revealed by SCXC.

**Fig. 1:**
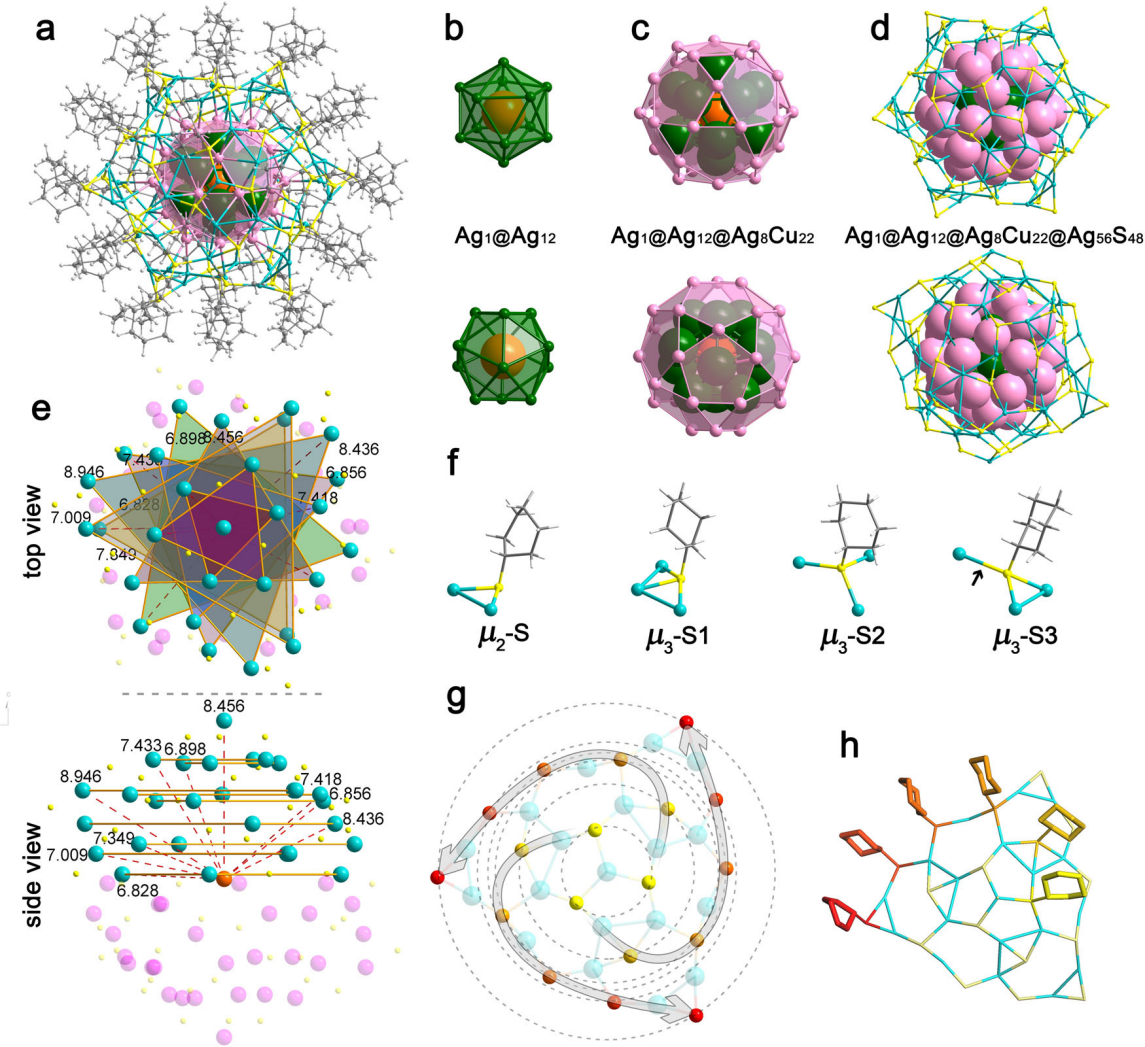
Crystal structure of Ag_77_Cu_22_(CHT)_48_. **a** Top view along the *C*_3_-axis of Ag_77_Cu_22_(CHT)_48_. Top and side views of each shell: **b** Ag_12_ shell, **c** Ag_8_Cu_22_ shell, **d** Ag_56_S_48_ shell. **e** The radial distances from the center Ag atom (orange) to the Ag atoms (cyan) on the outer shell. **f** The interface bonding motifs for AgCHT and **g**, **h** the ripple-like pattern of S atoms in Ag_19_S_18_ units. The colors of the S atoms change from yellow to red with ripple diffusion. The distorted arrows indicate the rotation of the helical stripes. Color codes: orange, green, cyan, pale pink, purple, Ag; pale pink, Cu; gray, C; light gray, H; others, S.

The crystal adopts a trigonal space group of *P*−3, and the 99 metal atoms are distributed in four shells peripherally protected by 48 CHT ligands (Supplementary Fig. 9 and Fig. 1a). Since the dissociation energy of the Cu−Cu bond is higher than that of the Ag−Ag or Ag−Cu bond, Cu atoms generally prefer to form a pure shell without enclosing Ag atoms in AgCu alloy NCs. Herein, we found that 22 copper atoms and eight silver atoms are distributed in the second outermost of the nanoparticle, probably because the quantity of Cu atoms in Ag_77_Cu_22_ is not enough to form a separate shell. The other three shells are fully occupied by silver atoms, and the nanoparticle has the structure of Ag_1_@Ag_12_@M_30_@Ag_56_(SR)_48_, as illustrated in Fig. 1b–e (the carbon and hydrogen atoms are omitted for clarity). The central Ag atom and the second shell form a conventional 13-atom icosahedron (Fig. 1b), which is enclosed by a 30-atom icosidodecahedron. The icosidodecahedron shell can be described as 20 triangular faces and 12 pentagonal faces joined together (see Fig. 1c), and every center of the pentagonal face corresponds to each vertex of the 12-atom icosahedron (Fig. 1c). The outermost shell consists of 56 Ag atoms and 48 S atoms with three gradients viewed from the side (Supplementary Fig. 10). Gradient 1 shows a dendritic growth mode to form a cone-like shape that is composed of 19 Ag atoms and 18 S atoms (Ag_19_S_18_ for short). Gradient 3 is the inverted shape of gradient 1, which appears as a mutual mirror from the top view. Six Ag_3_S_2_ units constitute gradient 2, which sews up gradients 1 and 3. Ten sets of equivalent Ag-atom sites are found in the half shell via the measurement of the radial distances and marked by nine triangles and one Ag on the apex (Fig. 1e). The other half (purple) has the same Ag-atom sites and thus is not marked. 24 atoms in the (AgCu)_30_ shell are coordinated to the Ag atoms in the Ag_56_S_48_ outer shell (every Ag or Cu atom in the (AgCu)_30_ shell bonds 2 or 3 Ag atoms in the Ag_56_S_48_ outer shell) except for the six metal atoms closed to the six vertexes (Supplementary Fig. 11). This shell can also be described as a combination of 36 distorted cyclic staples through edge- or vertex-sharing, including one type of Ag_3_S_3_ (1 × 6) staple and five types of Ag_4_S_4_ (5 × 6) staples (see Supplementary Fig. 12). These cyclic staples, with Ag and S atoms arranged alternately, are pieced together like different patches. Ag_3_S_3_ staples are located around the vertex of the gradient 1 or 3 cone, and two types of Ag_4_S_4_ staples (b, c in Supplementary Fig. 12) surround the Ag_3_S_3_ staples in a stepwise manner to form the Ag_19_S_18_ cone. Gradient 2 consists of the other three Ag_4_S_4_ staples (d, e, f in Supplementary Fig. 12). Ag_3_S_3_ staples are planar structures, while Ag_4_S_4_ staples are all distorted with zigzag or boat shapes. Generally, the S atoms in the staples protrude from the surface of thiolated nanoparticles, and, thus, can be viewed as a protected shell. However, the S atoms in the Ag_56_S_48_ shell almost lie in the same planes as the Ag atoms. In other words, S atoms are interlaced with Ag atoms in this shell except for the six vertices S (red atoms in Supplementary Fig. 10). This can also be proven by the fact that 73% of the Ag–S–Ag angle on the Ag_56_S_48_ shell exceeds 90°, and that such a ratio is the highest among the investigated thiolated nanoparticles (nanoclusters) in the crystal phase (Supplementary Fig. 13), probably due to the strong repulsion from the especially close nanoparticles (vide infra).

### Surface morphology

The surface motifs consist of 48 thiolates, including six two-coordinated and 42 three-coordinated thiolates, with an average Ag–S bond length of 2.4125 Å. *μ*_3_-S can be further categorized into three classes (*μ*_3_-S1, *μ*_3_-S2, and *μ*_3_-S3) in terms of the Ag–S–Ag angle (Fig. 1f). The thiolate ligand in the *μ*_3_-S1 type binds to the shell deviating from the Ag_3_ unit with small Ag–S–Ag angles, while the thiolate ligands in the *μ*_3_-S2 and *μ*_3_-S3 types bind to the Ag_3_ unit at relatively large angles. The difference between the *μ*_3_-S2 and *μ*_3_-S3 types is that the three Ag–S–Ag angles in *μ*_3_-S2 are all approximately equal to 110°, while one of the three angles in *μ*_3_-S3 turns to ca. 81°. It is worth noting that with the angle decreasing in *μ*_3_-S3, one Ag–S bond (indicated by a black arrow) is elongated to 2.835 Å, which is obviously longer than the common Ag–S bond (2.3–2.7 Å) (see the statistics in Supplementary Fig. 14) and rarely found in Ag nanoparticles (nanoclusters). Interestingly, a ripple-like arrangement was found for the Ag_19_S_18_ cone (gradient 1) in the outermost shell of Ag_77_Cu_22_, which has not been reported previously in silver nanoparticles (nanoclusters). Every three S atoms are assembled into a ring with one Ag atom at the center, leading to a ripple-like pattern with six rings (the six groups of S atoms are gradually marked from yellow to red, see Fig. 1g). Moreover, the S atoms, as well as the cyclohexyl tails with varying orientations (Fig. 1h), adopt a helical anticlockwise stripe pattern along the six rings. The same pattern is also observed on the opposite pole (gradient 3) but with a clockwise rotation.

**Fig. 2:**
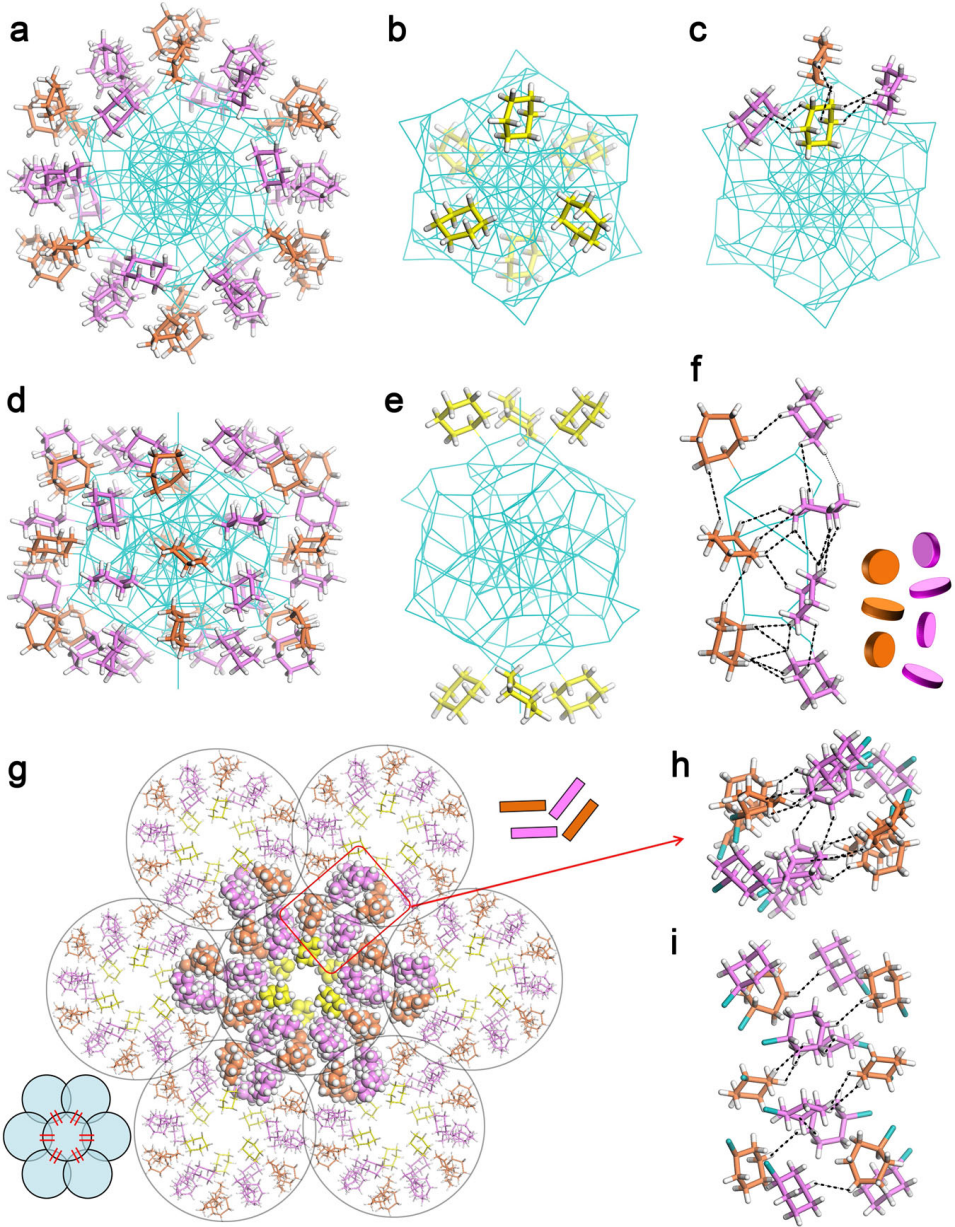
Surface pattern of the ligands on Ag_77_Cu_22_ NCs. The ligands are distributed perpendicular to the waist (**a**) or on the poles (**b**). **c** Interactions between the pole ligands and their surrounding ligands. **d**, **e** side views of **a** and **b**. **f** Interactions between the ligands perpendicular to the waist. **g** Seven close-packed Ag_77_Cu_22_ NCs with interactions in six directions. **h** Top view and **i** side view of the interparticle interactions point-by-point. Color codes: orange, pink, yellow, C; white, H; cyan, S, Ag, Cu.

Recently, hierarchically ordered patterns formed by the self-assembly of Au–SR on the surface of NCs have attracted extensive interest in manipulating property-related complex structures^43^. Here, the 48 ligands can be further divided into three sorts (Fig. 2a, b): six ligands (yellow in Fig. 2b) are equally distributed into two groups on the poles with ca. 60° rotation; 24 ligands in six groups (pink in Fig. 2a) are arranged on the surface perpendicular to the waist; and the remaining 18 ligands (orange in Fig. 2a) are aligned into six parallel groups to form an alternating pattern with the abovementioned 24 ligands (like a clock dial). A complete H···H interaction network is formed inside the Ag_77_Cu_22_ nanoparticle. For example, every ligand (yellow) around the poles shows H···H interactions with three adjacent ligands (Fig. 2c), and the 12 groups of ligands across the waist also show H···H interactions in two dimensions (see Fig. 2d–f, H···H spacing in Supplementary Table 1). Notably, the H···H spacing across the waist is very short (average value of 2.714 Å), which can influence the formation of interlocked ligands via the cyclohexane orientation. When the two neighboring groups (including seven ligands) are regarded as one unit, it is found that every cluster connects with the surrounding clusters through the six abovementioned units (Fig. 2g). As shown in Fig. 2h, i and Supplementary Table 1, the H···H interactions between clusters intersect between four contacting groups of ligands, indicating dense packing among clusters.

### Polymeric structure

**Fig. 3:**
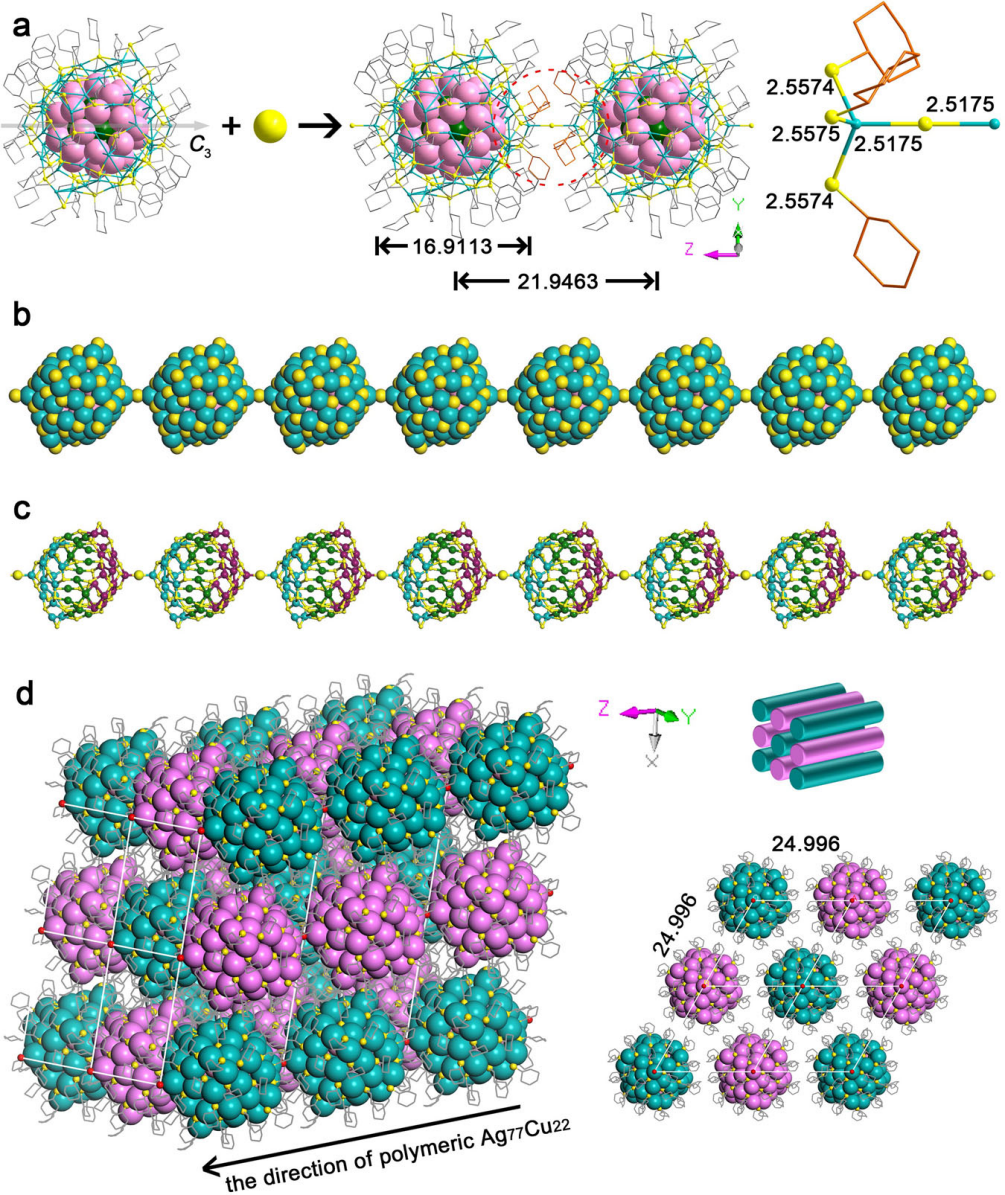
Polymeric structure of Ag_77_Cu_22_ NCs. **a** The linkage of Ag_77_Cu_22_ NCs (H atoms are omitted for clarity). **b** Space-filling view of the Ag_77_Cu_22_ chain (C and H atoms are omitted for clarity). **c** The arrangement of Ag_56_S_48_ shells along the Ag_77_Cu_22_ chain (C and H atoms are omitted for clarity). **d** 3D framework of Ag_77_Cu_22_ nanowires and view of the superlattice from the *z*-axis (note that the two-color scheme in Fig. 3d does not indicate the chirality, but means different chains in the crystal packing). H atoms are omitted for clarity. The length unit is Å. Color codes: cyan, green, pink, purple, Ag; pale pink, Cu; yellow, red, S; gray, orange, C.

Another unusual feature of the crystal structure is the straight S-linked nanoparticle chains along the *z*-axis (Fig. 3) formed by connecting the pole Ag atoms with S ions. The linker “X” can be “S”, “SH”, “Cl”, or “Br” on the basis of the starting materials used in this reaction, however, both X-ray photoelectron spectroscopy (XPS) (Supplementary Fig. 15) and energy dispersive spectrometer (EDX) (Supplementary Fig. 8) exclude the existence of Cl or Br. Ion chromatography (IC) and precipitation experiments (PEs) further exclude the existence of Cl^–^ or Br^–^, and identify the “X” to be “S^2–^” (Supplementary Fig. 16). The strong alkaline reaction solution (NaBH_4_) does not prefer the forming of HS^–^, too. IR spectrum further excludes the existence of HS^–^ since the feature signal of HS^–^ at ~2550 cm^–1^ was not found in the IR spectrum (Supplementary Fig. 17). The bridging S^2–^ ions are probably produced from the C–S bond cleavage of thiol or thiolate, as was studied by us^44^ and some other groups^45–48^. As shown in Fig. 3a, b, the Ag–S–Ag angle formed by the two pole silver atoms and the linker S is equal to exactly 180° and the radial direction of such chains coincides with the *C*_3_-axis of every single Ag_77_Cu_22_ NC, perpendicular to the *x*–*y* plane in the crystal packing. Therefore, the chains are perfectly straight lines without any rotation of the NCs. Note that to make room for the S linker, the three thiolates around the Ag pole stretch out compared with other ligands, forming a “hole” or an “open” Ag for the linkage (Fig. 3a). As shown in Fig. 3c, the two mirrored Ag_19_S_18_ cones on the shell marked with cyan and purple are also arranged alternately in the chains. The lengths of the Ag–S bonds for every sulfur atom linking two NCs are both equal to 2.5175 Å, which is even shorter than that of some typical Ag–S bonds (2.356–2.835 Å) in the shell. The metal core diameter of Ag_77_Cu_22_ is ca. 1.69 nm, and the interparticle distance along the chain is ca. 2.19 nm (Fig. 3a). However, the interparticle distances along the *x*- and *y*-axes are both ca. 2.5 nm (Fig. 3d), implying a stronger interaction between the neighboring nanoparticles along the chain direction, as also demonstrated by the inter-nanoparticle H···H interactions. As shown in Supplementary Fig. 18, the close inter-nanoparticle H···H spacings, such as 2.288, 2.561, and 2.642 Å, are observed along the chain direction, while the shortest inter-nanoparticle H···H spacing along the perpendicular direction (*x*- and *y*-axes) is 2.422 Å (Supplementary Table 1).

### Magnetism performance

**Fig. 4:**
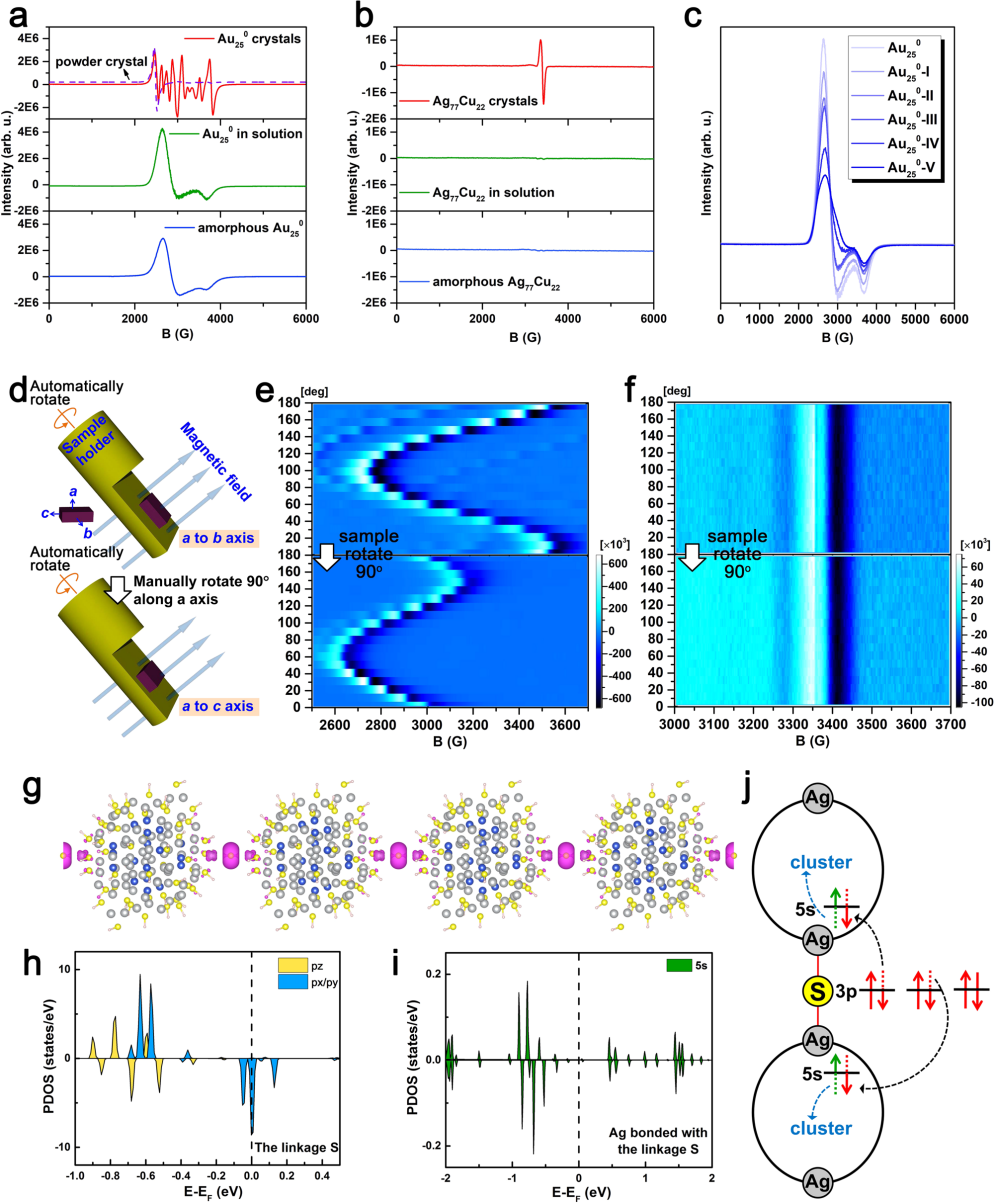
Magnetic performance of Ag_77_Cu_22_ and Au_25_^0^. EPR spectra for **a** Au_25_^0^ and **b** Ag_77_Cu_22_ in different states (note: the solution samples were aged for 1 h before measurements, and the amorphous samples were prepared from the above-aged solutions by fast precipitation and drying). **c** EPR spectra measured for Au_25_^0^ NCs with different CHT substitution numbers. **d** Schematic depiction of the rotational EPR experiment. 2D EPR density plots of **e** Au_25_^0^ and **f** Ag_77_Cu_22_. **g** Spin distribution of polymeric Ag_77_Cu_22_ (magenta: spin density). Spin-polarized PDOS of the **h** *3p* orbitals of the S linkage and **i** *5* *s* orbitals of Ag bonded with the S linkage. **j** Schematic representation of the electronic structures of the linkage.

The nominal shell-closing electron count (n*) for the Ag_77_Cu_22_ nanoparticle is 49 (n* = *Nv*_A_ − *M* − *z* = 77 × 1 + 22 × 1 – 48 − 2 = 49)^49^, reminding one of its paramagnetism^50^, which was confirmed by experiments. For comparison, the paramagnetism of neutral Au_25_ was also investigated. As shown in Fig. 4a, the EPR spectrum for a small amount of neutral Au_25_ single crystals shows multiple peaks (red curve). When the large crystals are broken into microcrystals by ultrasonication, the refined peaks disappear, and broadband (identical to the reported spectrum^51,52^) appears, indicating the influence of the crystal orientation and the magnetic anisotropy of the Au_25_ crystal. The EPR measurement was conducted by automatically or manually rotating the single crystal sample (illustrated in Fig. 4d) to further probe the magnetic anisotropy. As shown in Fig. 4e, the intensity and position of the EPR signals for the Au_25_ single crystal change stepwise as the sample is rotated, further confirming the magnetic anisotropy of neutral Au_25_ crystals. In contrast, the Ag_77_Cu_22_ nanoparticle assembly shows a different EPR spectrum from that of a single Au_25_^0^ crystal (Fig. 4b and Supplementary Fig. 19), indicating different origins for the magnetism. The blank experiments exclude the external introduction of magnetic impurities, see Supplementary Fig. 20. The simulated results (*g* = 2.0072, 1.9671, and 1.9659) are well consistent with the experimental observations (Supplementary Fig. 21). Particularly and interestingly, the EPR spectra remain the same after rotating the single crystal sample (Fig. 4f), indicating magnetic isotropy. Such magnetic isotropy is unusual and has rarely been reported in metal nanoparticles^53,54^, which inspires our enthusiasm for further investigation. Due to the difficulty of experimentally mapping the magnetism resource, DFT calculations were performed to acquire the spin distribution using the crystal structure with simplified ligands to reduce the computational cost^55^. The crystal structure is preserved (other than replacing R=cyclohexyl groups by R=H-atoms), and the collinearity of *C*_3_, the linker, and the *z*-axis is preserved when relaxed, see Supplementary Fig. 22. The spin density distribution is shown in Fig. 4g. Remarkably, the magnetic moment is found to be mainly localized at the S^2–^ linker rather than the metal atoms (Ag or Cu). Only weak spin density is revealed on the surface or kernel atoms adjacent to S^2–^, and the magnetic moment of the linker S^2–^ is almost 12 times larger than that of the Ag atom with the largest magnetic moment. The detailed spin distribution is given in Supplementary Table 2. In strong contrast, the magnetic moment of Au_25_ is found to be mainly localized at the kernel Au atom (see Supplementary Fig. 23 and Supplementary Table 3), similar to the results reported by ref. 50. It is generally believed that magnetic anisotropy has diverse origins^53,56^, such as spin–orbit coupling (SOC), exchange interaction, etc. For Au_25_, the magnetic anisotropy can be attributed to the strong SOC of Au atoms since the noncollinear calculation reveals large magnetic anisotropy energy (MAE, ca. 200 μeV). The torque method was further applied for analysis^57^, and it was revealed that the positive uu (27.78 meV) and dd (27.96 meV) channels contribute more (97 μeV) than the negative ud + du (–55.65 meV) channel, leading to a positive MAE, which provides further support for the SOC mechanism for the magnetic anisotropy. For the Ag_77_Cu_22_ assembly, given the consideration of the major spin on S^2–^, the SOC mechanism is not applicable since the SOC parameter for S (288 cm^–1^) with a relatively small atomic number is much lower than that for Cu (1150 cm^–1^) or Ag (3540 cm^–1^)^58^, and, thus, the SOC effect is negligible; the exchange interaction can also be excluded given the large distances between S spins (2.2 nm along the chain direction; 2.5 nm perpendicular to the chain direction); magnetic measurements using a Quantum Design MPMS3 SQUID magnetometer were used to also exclude the magnetic interaction since the Curie-Weiss temperature obtained by fitting variable temperature magnetic susceptibility was close to 0 (see Supplementary Fig. 24), implying an absence of spin interaction. The temperature-dependent EPR further confirms the noninteraction of the spins, see Supplementary Fig. 25. The magnetic isotropy of the assembly can be ascribed to the major spin on sulfur confined in such a special structure. Note that the *g*-factor and specific magnetization (emu/g) are shown in Supplementary Figs. 21 and 24, respectively. The *g*-value (1.9790) was close to 2.0023 (*g*-value of a free electron) and the obtained effective moment *μ*_eff_ = 1.62 *μ*_B_ is also consistent with *S* = ½ per cluster molecule (1.73 *μ*_B_), supporting the notion that the magnetism of Ag_77_Cu_22_ assembly originates from the unpaired shell electron of the Ag_77_Cu_22_ nanoparticle. Notably, the hyperfine splitting (fine structure) was not observed in the EPR spectra, which might be interpreted by the delocalization^33,50,59,60^ of the unpaired spin or the weak spin–nuclei interaction due to the extremely low abundance of magnetic ^33^S nuclei^61,62^. A natural question arises: why the magnetic moment mainly locates at S^2–^. It is possible that electron transfer occurs from linker S^2–^ to the metal atom. Such electron transfer between sulfur and the nanoparticle core was revealed in our previous photoluminescence study^63^. The close linker (Ag–S distance) also provides support for such a phenomenon. A study of the projected density of states (PDOS) revealed that the linker S shows a magnetic moment of ~1 *μ*_B_ with half-occupied spin-down states of *p*_*x*_/*p*_*y*_ orbitals, while the connected Ag exhibits a very small moment with partially occupied spin-up and spin-down states of *5s* orbitals (see Fig. 4h, i), providing further support for the above proposal. Considering the bonding of every S^2–^ linker with two nanoparticles, we suggest that the electron transfer for S^2–^ is divided between the two connected silver atoms (Fig. 4j), forming one unpaired spin, and the electron on Ag can further transfer to the neighboring atoms, resulting in a distribution of the magnetic moment for the nanoparticle (Fig. 4g and Supplementary Table 2). The *g* = 1.9790 determined from the EPR spectrum (Supplementary Fig. 21) reminds one of the existence of sulfur radicals^64^. To further verify the electron transfer from sulfur to the connected silver (i.e., formation of the sulfur radical), DMPO was used to capture the radical. As shown in Supplementary Figs. 26 and 27, strong radical signals were observed for the case of Ag_77_Cu_22_ assembly, but no significant signals were detected for the case of Au_25_, confirming the presence of sulfur radicals in the Ag_77_Cu_22_ assembly and the absence of sulfur radicals in Au_25_, since that the sulfur radicals can be captured by DMPO and lead to the emergence of the EPR signal of the trap adducts as well as the decrease of the paramagnetism signal.

Although the monosulfur radical signal was once detected under some specific conditions, the precise structure involving monosulfur radicals was undocumented^64–66^. Herein, the active sulfur radical might be stabilized by the assembly structure, which was confirmed by subsequent experimental results. When the assembled crystals are dissolved in dichloromethane, the EPR intensity decreases with increasing time (see the time evolution of the integrated EPR intensity in Supplementary Fig. 28 and the green line in Fig. 4b), and the EPR signal remains weak in the amorphous state after the solvent is removed (see blue line in Fig. 4b). In contrast, the EPR intensity for neutral Au_25_ crystals is not quite influenced by dissolution (green line in Fig. 4a). Taken together, these facts demonstrate that the sulfur radical is stabilized by the specific assembly structure, which has important implications for novel functional development based on the assembly of atomically precise nanoparticles. It is concluded that chain structure disassembly can lead to electron return and the recovery of S^2–^, which was verified by ion chromatography: no other sulfur ion species except for S^2–^ is detected in the extract of the disassembly solution (Supplementary Fig. 16b). After the electron returns to sulfur, the Ag_77_Cu_22_ nanoparticle is expected to bear an unpaired shell-closing electron, and thus, exhibits paramagnetism in solution (Supplementary Fig. 29). However, unlike the neutral Au_25_ nanocluster, Ag_77_Cu_22_ shows a decreasing EPR intensity with increasing time in the solution, as mentioned above. A plausible reason for this is the spin pairing of Ag_77_Cu_22_ nanoparticles due to the short and flexible ligands that enable the nanoparticles to approach each other. However, the Au_25_ nanoclusters cannot couple in solution due to the interference of the relatively long and rigid ligands, which is verified by TEM images since nanoparticle pairs are found for Ag_77_Cu_22_ but not for 2-phenylethanethiolated Au_25_, as shown in Supplementary Figs. 30, 31a. Previous work also provides support: polymeric Au_25_ capped by *n*-butanethiolates was proven to be antiferromagnetic due to spin coupling along the polymer chain^33^. Au_25_ clusters along the chain (*z*-direction) are connected directly via Au−Au interactions, and the interparticle distance is 1.3001 nm, which is significantly shorter than that in the *x*- and *y*-directions (1.6131 nm and 1.7982 nm, respectively). To further probe the influence of the ligand on nanoparticle coupling, we conducted an EPR test for neutral Au_25_ protected by a stiffer ligand (naphthalenethiolate, Nap) or a more flexible ligand (CHT) than 2-phenylethanethiolate (PET). Note that the 2-phenylethanethiolates on the Au_25_ nanocluster are fully exchanged by naphthalenethiolates but not by cyclohexanethiolates, as identified by SCXC or mass spectra (Supplementary Figs. 32 and 33), and five samples with varying content of cyclohexanethiolates were prepared. All five samples share UV−Vis spectra that are similar to that of Au_25_(PET)_18_^0^ (Supplementary Fig. 34), implying structural similarity with Au_25_(PET)_18_^0^. The substitution numbers (Xs) were estimated from ESI-MS as follows: Au_25_-I, x = 2–4; Au_25_-II, x = 3–6; Au_25_-III, x = 5–9; Au_25_-IV, x = 11–15; Au_25_-V, x = 13–17 (Supplementary Fig. 33). The experimental results reveal that naphthalenethiolated Au_25_ shows similar EPR signals in both solution and crystals without the time-dependent intensity observed in solution (Supplementary Fig. 35), while cyclohexanethiolated Au_25_ exhibits cyclohexanethiolate content and a time-dependent EPR intensity in solution (Fig. 4c and Supplementary Fig. 36). Furthermore, the decrease in the EPR intensity corresponds to the nanoparticle pairing degree observed in TEM images (that is, the larger the decrease in the EPR intensity, the denser nanoparticle pairing, see Supplementary Fig. 31). Similarly, Maran et al. previously revealed that the EPR intensity of ethanethiolated Au_25_ is influenced more by its state (crystal or solution) than n-butanethiolated Au_25_^33^. Taken together, these facts demonstrate that the inter-nanoparticle distance (M: metal atom) is critical for spin coupling (i.e., spin coupling only occurs when the inter-nanoparticle distance is short enough). It is worth noting that such spin coupling does not result in the growth of nanoparticles since no large nanoparticles (or dimers) were detected by ESI-MS, UV-Vis spectrometry, and TEM (see Supplementary Figs. 37 and 38).

## Discussion

In summary, we introduced an on-site synthesis-and-assembly strategy to solve the challenge of assembling atomically precise metal nanoparticles. A straight-chain structure composed of [Ag_77_Cu_22_(CHT)_48_]^2+^ nanoparticles and linker S^2–^ was obtained, and it was precisely characterized by using ESI-MS and SCXC. Interestingly, a rare Ag/Cu alloy shell was observed in the nanoparticles, and cyclic staples and a ripple-like thiolate distribution on the nanoparticles were, to the best of our knowledge, also reported for the first time. In particular, both calculations and experiments such as EPR and DMPO probing were used to reveal that the linker is sulfur radical with magnetic isotropy, which is formed by *2p* electron transfer from S^2–^ to the connected silver atoms and stabilized by the chain structure. The disassembly of the straight-chain structure in solution leads to the return of the electron to S^2–^ and restoration of the paramagnetism of the Ag_77_Cu_22_ nanoparticles. However, the EPR intensity for Ag_77_Cu_22_ decreases with increasing time, which is ascribed to the spin coupling of Ag_77_Cu_22_ nanoparticles in solution. Further experiments reveal that the inter-nanoparticle distance is critical to the nanoparticle (nanocluster) spin coupling (i.e., coupling only occurs when the inter-M-M distance is short enough). Such an assembly-induced spin transfer and inter-nanoparticle distance-dependent spin coupling are not only interesting and surprising but also have important implications for the novel functional development of nanoparticles and the understanding of the shell-closing electron behavior of superatoms. It is expected that our work will extend a promising but still challenging field−the assembly of metal nanoparticles with atomic precision.

## Methods

### Chemicals

All chemicals were commercially available and used as received. Silver nitrate (AgNO_3_, 99.99%), cuprous chloride (CuCl, 95%), cyclohexanethiol (CHT, 98%), tetraphenylphosphonium bromide (TPPB, 98%), naphthalenethiol (Nap, 99%), 2-phenylethanethiol (97%), and sodium borohydride (NaBH_4_, 98.0%) were purchased from Aladdin. 5, 5-Dimethyl-1-pyrroline *N*-oxide (DMPO) was obtained from Dojindo. Triethylamine (99%), ethanol (AR), dichloromethane (AR), hexane (AR), Toluene (AR), and ethanol (AR) were purchased from Sinopharm chemical reagent co., ltd.

### Synthesis of AgCu nanoclusters

AgCHT complexes were prepared by mixing 120 mg of AgNO_3_ and 200 μl of CHT in MeOH. Then, 22 mg of the washed AgCHT complexes and 10 mg of CuCl were dissolved in 40 ml of CH_2_Cl_2_ in a 100 ml of single-neck round-bottom flask under vigorous stirring. Then, 20 μl of triethylamine was immediately added to the reaction solution. After that, 4 ml of an ethanol solution of NaBH_4_ (38 mg) and TPPB (42 mg) was added dropwise to the mixture, which showed a color change from yellow to dark brown. The reaction was then kept under continuous stirring for 6 h under ice-cold conditions in the absence of light. The mixture was rotary evaporated to dryness to give a dark solid, which was washed with methanol three times. The as-obtained precipitate was extracted using 15 ml of CH_2_Cl_2,_ and the supernatant was aged for over a week at room temperature to form a green solution. Finally, a concentrated green solution (0.5 ml) was placed into a clean NMR tube layered with ca. 2 ml of hexane. Then, the tube was carefully capped and stored in a refrigerator at 4 °C. Black crystals were obtained after approximately 1 month.

### Ligand exchange of Au_25_(PET)_18_

Neutral Au_25_ NCs were first prepared according to the previous literature:^67,68^ 10 mg Au_25_ anions were dissolved in 8 ml CH_2_Cl_2_ and treated with 200 μl H_2_O_2_ (30%) for 20 min at room temperature, then the organic solution was extracted and purified using preparative thin-layer chromatography to obtain Au_25_^0^ NCs. Six milligrams of neutral Au_25_ was dissolved in 300 μl of toluene and then reacted with different amounts of CHT at room temperature for 30 min or 60 min. The products were precipitated and washed with methanol. Au_25_(Nap)_18_ was synthesized at 70° by mixing neutral Au_25_ with excess Nap. The final product was purified using preparative thin-layer chromatography^69–71^.

### Characterization

Optical absorption spectra were acquired in the wavelength range of 190–900 nm using a Shimadzu UV-2600 spectrophotometer. Electrospray ionization mass spectrometry (ESI-MS) was performed on a Waters Q-TOF mass spectrometer equipped with a Z-spray source. Single crystal X-ray diffraction data were collected on a Bruker D8 Venture X-ray diffractometer (Bruker, Germany). The possible reasons for the high residual density are the inadequate absorption correction of silver which strongly absorbs X-rays or the uncertainty of the metal atoms (Ag or Cu), resulting in unreal residual Q peaks. The low C-C bond precision originates from the restraints on the hexyl ligands (S2 and S5) due to their varied configuration and weak diffraction intensity. X-band CW-EPR spectra were collected using a Bruker EMX plus 10/12 X-band EPR spectrometer equipped with an ER4119HS (TE011) high-Q cavity. Cryogenic temperatures were achieved using an Oxford Instruments ESR910 liquid helium flow cryostat and an Oxford Instruments ITC503 temperature controller. A programmable one-axis goniometer was used for the sample rotation. Transmission electron microscopy (TEM) images were recorded using a Tecnai G2 TF20 instrument (FEI Co., USA). Matrix-assisted laser desorption ionization mass spectrometry (MALDI-MS) was performed on an Autoflex Speed TOF/TOF mass spectrometer (Bruker) in positive ionization mode. Magnetization measurements were carried out for the NCs (sealed into nonmagnetic capsules) as a function of temperature and field by using a SQUID magnetometer (Quantum Design MPMS3).

### Theoretical methods

Ab initio calculations were performed based on spin-polarized density functional theory (DFT), as implemented in the Vienna Ab initio Simulation Package (VASP)^72^. The ion-electron interaction was described by the projector augmented wave (PAW) approach^73,74^. The generalized gradient approximation (GGA) in the scheme of the Perdew-Burke-Ernzerhof (PBE) functional was used to describe the exchange-correlation interaction^75,76^. The force and energy convergence criteria used for the electronic structure calculations were less than 0.02 eV/Å and 10^–5^ eV, respectively. Given the consideration of the strong correlation effect on the d orbitals of Au, Ag, and Cu, GGA + U calculations with effective *U* values of 1.5 eV (Au) and 1 eV (Ag and Cu) were performed by including a Hubbard U term^77,78^. The spin moment and MAE were calculated by taking into consideration of spin-orbital coupling and magnetic noncollinearity^79^. EPR simulations were carried out by assuming a distribution of randomly oriented S = 1/2 spin centers with a Lorentzian lineshape using the EasySpin toolbox working on the MatLab software platform.

### Reporting summary

Further information on research design is available in the Nature Research Reporting Summary linked to this article.

## Supplementary information

Supplementary Information

Reporting Summary

## Data Availability

The X-ray crystallographic coordinates for structures reported in this work have been deposited at the Cambridge Crystallographic Data Center (CCDC) under deposition numbers 2207144 for the polymeric Ag_77_Cu_22_ nanoclusters, respectively. These data can be obtained free of charge from the CCDC via www.ccdc.cam.ac.uk/data_request/cif. Checkcif file for Ag_77_Cu_22_ CIF file is given as Supplementary Dataset. All data supporting the findings of this study are available within the article and its Supplementary Information files. Data were available from the first author and the corresponding author upon request.
