# Supplementary material for: Assembly-induced spin transfer and distance-dependent spin coupling in atomically precise AgCu nanoclusters
Source: Nat Commun. 2022 Oct 8;13:5934. doi: 10.1038/s41467-022-33651-9 (PMC9547874; doi:10.1038/s41467-022-33651-9)
Supplement: Supplementary file 1 — Supplementary Information [file 41467_2022_33651_MOESM1_ESM.pdf]

Supplementary Information

Assembly-induced spin transfer and distance-dependent spin coupling in atomically precise AgCu nanoclusters

Nan Xia[1,2,†], Jianpei Xing[3,†], Di Peng[1,4,†], Shiyu Ji[1,4], Jun Zha[1,4], Nan Yan[1,2], Yan Su[3], Xue Jiang[3], Zhi Zeng[1,*], Jijun Zhao[3,*], Zhikun Wu[1,2,*]

[1]Key Laboratory of Materials Physics, Anhui Key Laboratory of Nanomaterials and Nanotechnology, Institute of Solid State Physics, *HFIPS*, Chinese Academy of Sciences, Hefei 230031, P. R. China.

[2]Institute of Physical Science and Information Technology, Anhui University, Hefei 230601, P. R. China.

[3]Key Laboratory of Material Modification by Laser, Ion and Electron Beams (Dalian University of Technology), Ministry of Education, Dalian, 116024, P. R. China.

[4]University of Science and Technology of China, Hefei 230601, P. R. China.

[†]N. X., J. X, and D. P. contributed equally to this work

*Email: zkwu@issp.ac.cn, zhaojj@dlut.edu.cn, zzeng@theory.issp.ac.cn

Contents

Supplementary Figure 1. On-site synthesis-and-assembly strategy …………………………..3

Supplementary Figure 2. UV−Vis spectra ……………………………………………...3

Supplementary Figure 3−7. Mass spectra ……………………………………………3−6

Supplementary Figure 8. EDX analysis ……………………………………………………6

Supplementary Figure 9. Optical microscopy image ……………………………………6

Supplementary Figure 10−14. Analysis of the crystal structure ……………………………7−9

Supplementary Table 1. The distances of H···H interaction ……………………………………9

Supplementary Figure 15. XPS patterns…………………………………………………10

Supplementary Figure 16. Ion chromatograph ……………………………………………10

Supplementary Figure 17. Infrared spectrum ……………………………………………11

Supplementary Figure 18. H···H interaction on the linkage ……………………………11

Supplementary Figure 19−29, Table 2, 3. Magnetism measurements and calculations……12−17

Supplementary Figure 30, 31. TEM images ………………………………………………18

Supplementary Figure 32. Crystal structure of $Au_{25}(Nap)_{18}$ ……………………………19

Supplementary Figure 33. Mass spectra of CHT-substituted Au_{25}^{0} …………………………..19

Supplementary Figure 34. UV−Vis spectra of CHT-substituted Au_{25}^{0} ………………………20

Supplementary Figure 35. EPR spectra of $Au_{25}(Nap)_{18}$ …………………………………20

Supplementary Figure 36. Time evolution of the EPR spectra of Au_{25}-III ……………………21

Supplementary Figure 37, 38. Characterizations of disassembled $Ag_{77}Cu_{22}$ crystals ……21−22

Supplementary Table 4. Crystal data for $Ag_{77}Cu_{22}$ ……………………………………23

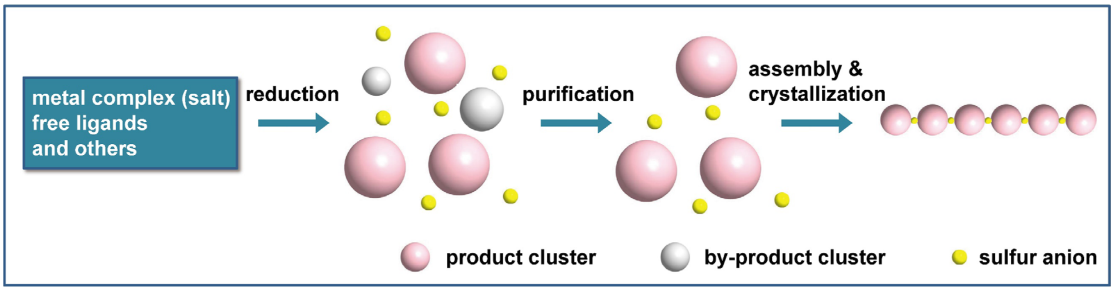

Supplementary Figure 1. On-site synthesis-and-assembly strategy.

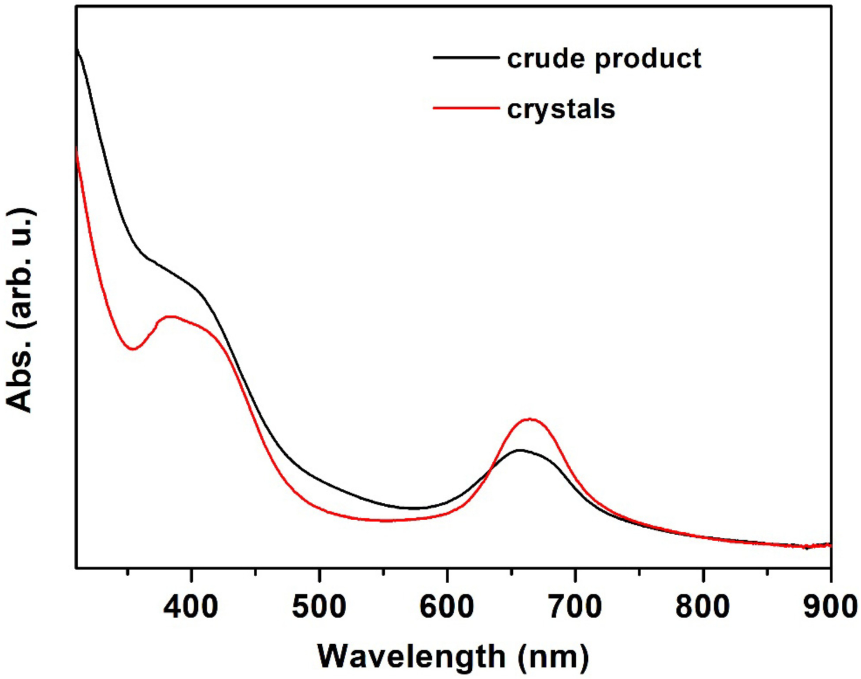

Supplementary Figure 2. UV−Vis spectra for the $Ag_{77}Cu_{22}$ in CH_2Cl_2 solution.

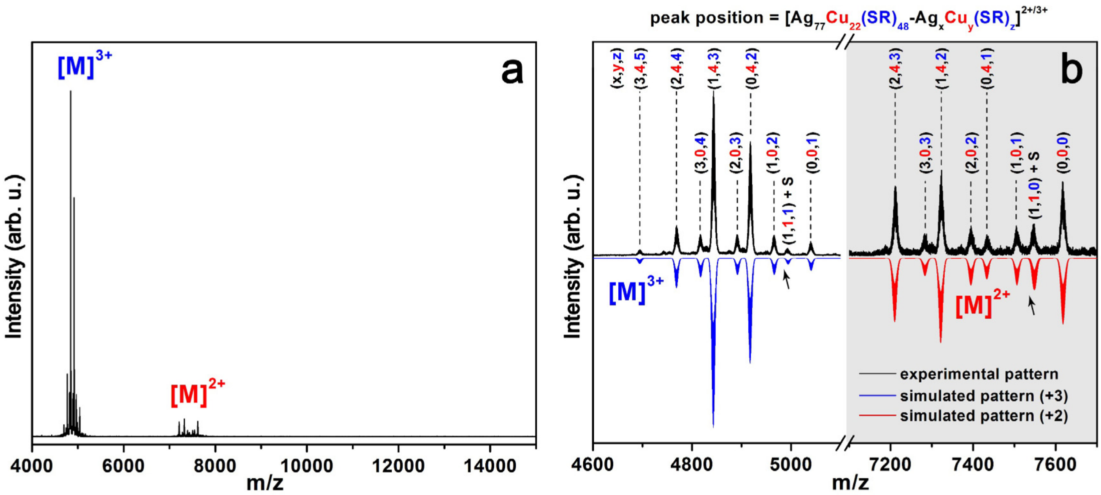

Supplementary Figure 3. (a) The full range of the ESI mass spectrum of $Ag_{77}Cu_{22}$ in the positive mode. (b) Mass peaks of $Ag_{77}Cu_{22}(CHT)_{48}$ in 3+ (red line) and 2+ (blue line) charged ions. The numbers shown on the peak labels correspond to the lost $Ag_xCu_y(SR)_z$ species, and the peak values are equal to the intact molecular weight minus the weight of the $Ag_xCu_y(SR)_z$ fragment.

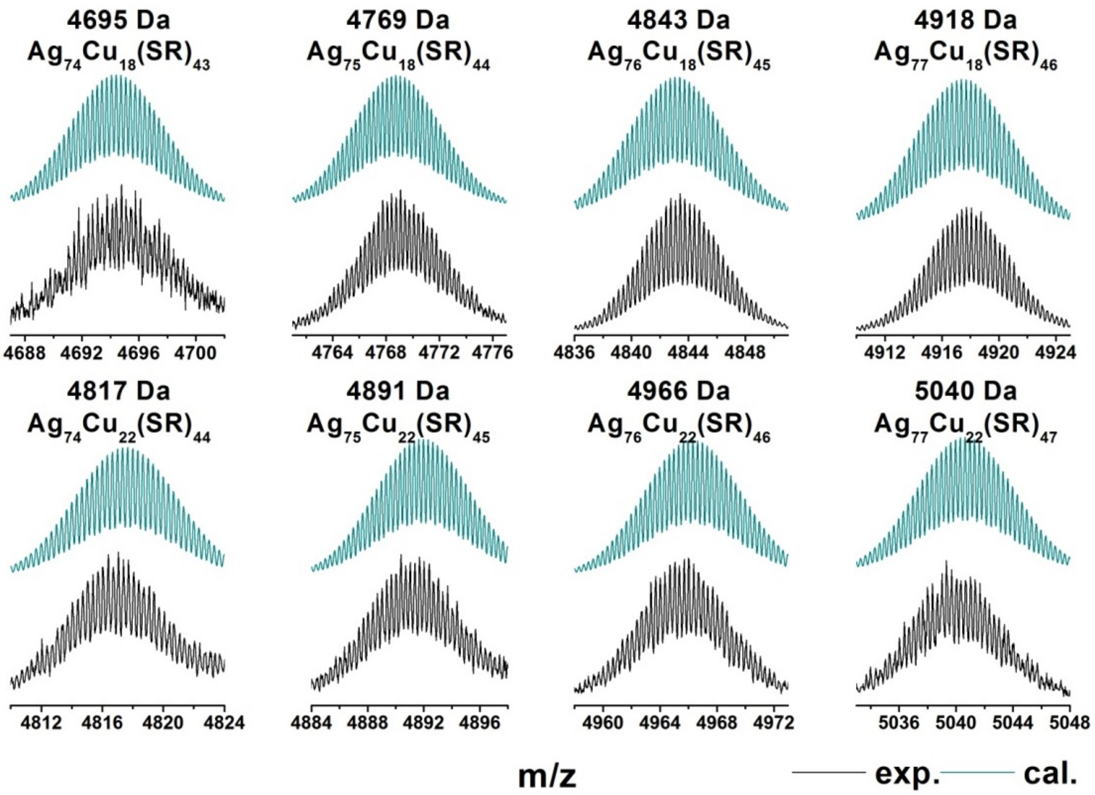

Supplementary Figure 4. The isotope distributions of the species bearing a charge of 3+. The cyan lines show the corresponding simulated isotope distribution.

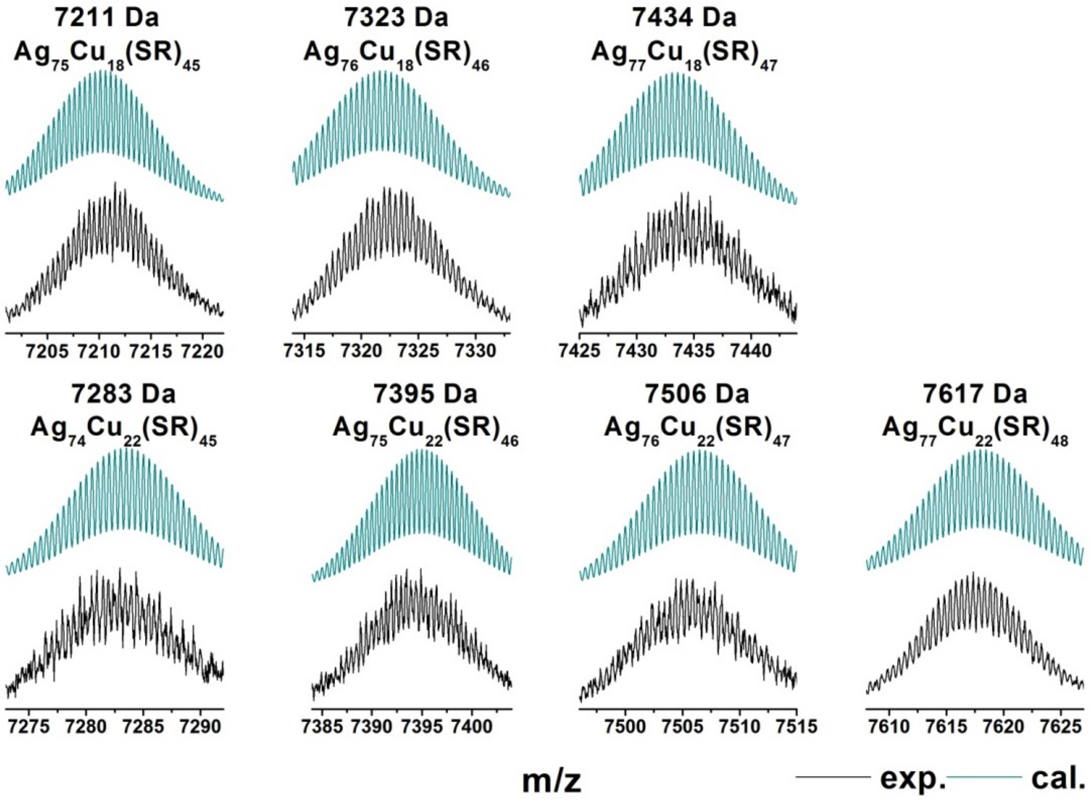

Supplementary Figure 5. The isotope distributions for the species bearing a charge of 2+. The cyan lines show the corresponding simulated isotope distribution.

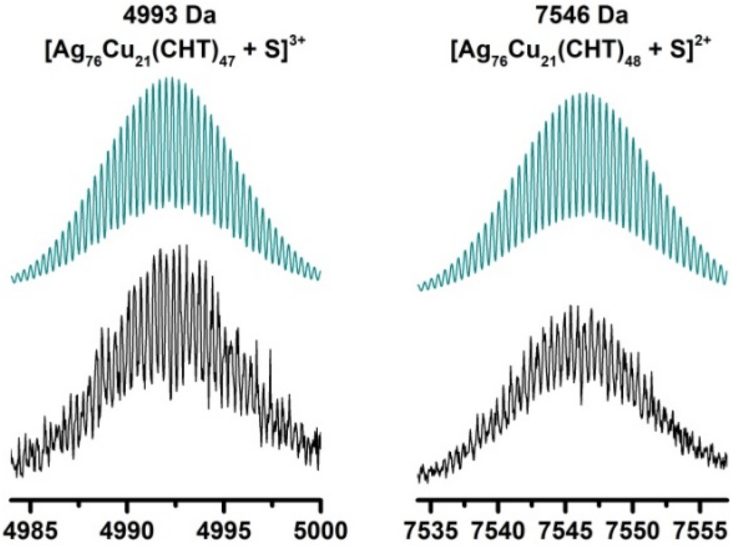

Supplementary Figure 6. The isotope distributions for species with added S atoms. The cyan lines show the corresponding simulated isotope distribution.

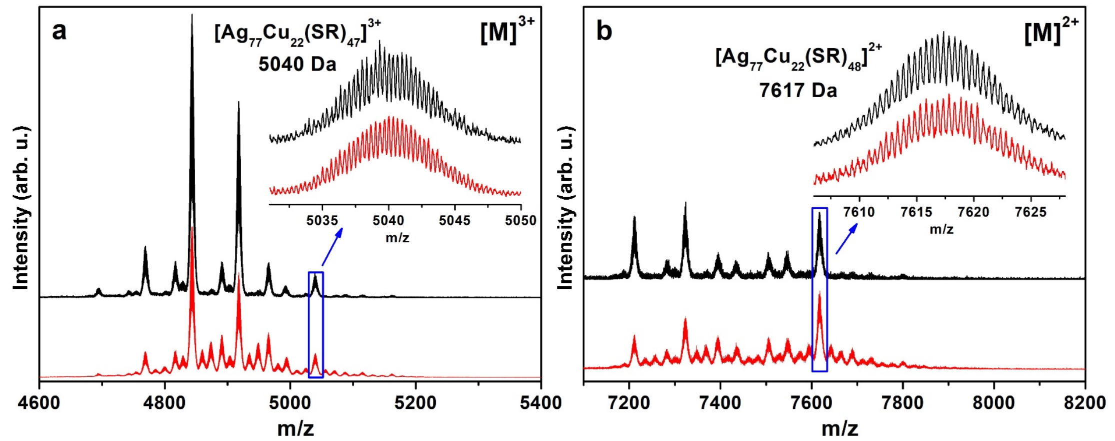

Supplementary Figure 7. The mass spectra of $Ag_{77}Cu_{22}$ nanoclusters reduced by $NaBH_4$ (black line) and $NaBD_4$ (red line): (a) +2 charge peaks, (b) +3 charge peaks. The insets are isotope distributions of +2 and +3 molecular ions marked by blue frame.

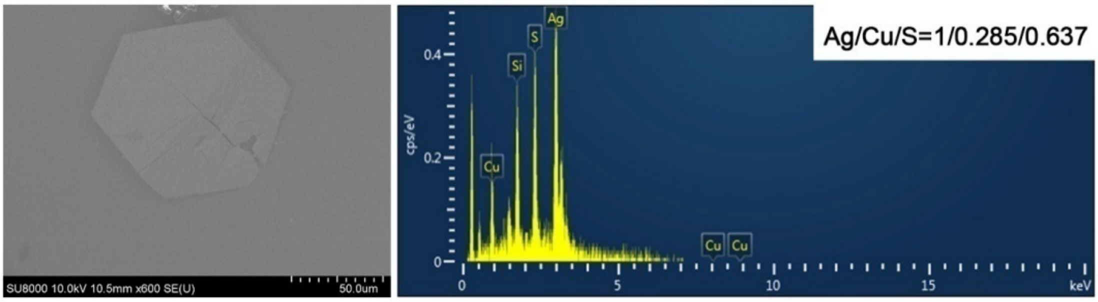

Supplementary Figure 8. Energy Dispersive Spectrometer (EDX) analysis of the $Ag_{77}Cu_{22}$ crystal.

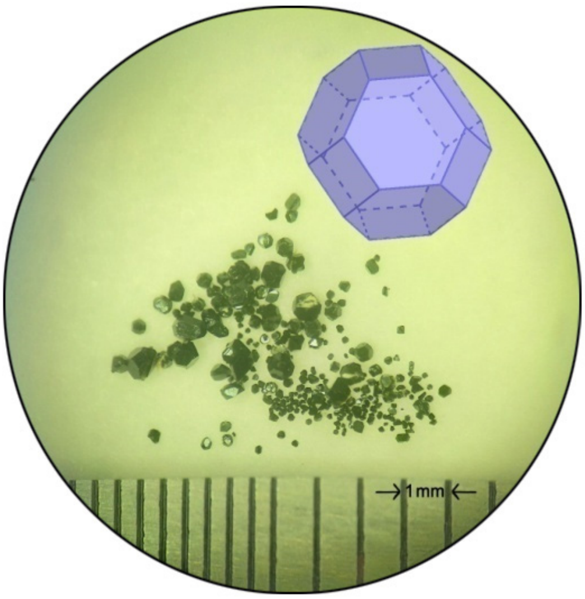

Supplementary Figure 9. Optical microscopy image of $Ag_{77}Cu_{22}$ crystals. Inset shows the truncated octahedron shape of the crystals.

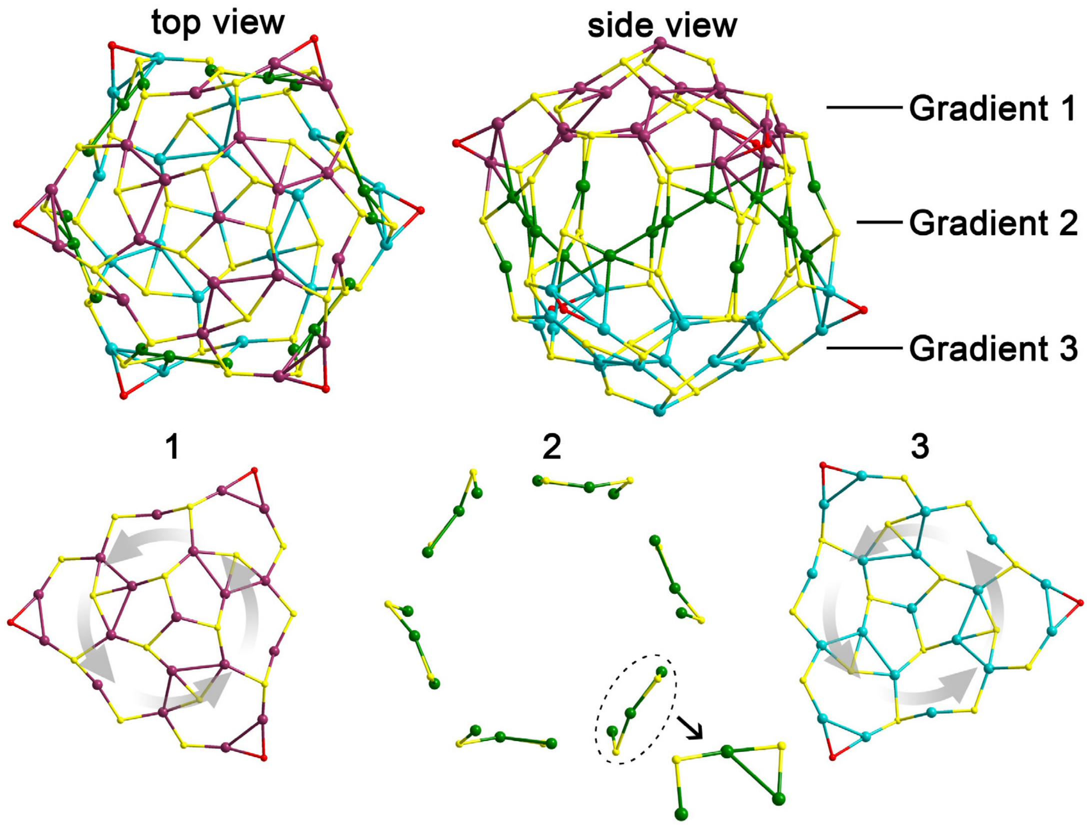

Supplementary Figure 10. Anatomy of the $Ag_{56}S_{48}$ shell: top view, side view, and the separated three gradients (1, 2, 3). Color codes: cyan, green, purple, Ag; yellow, red, S.

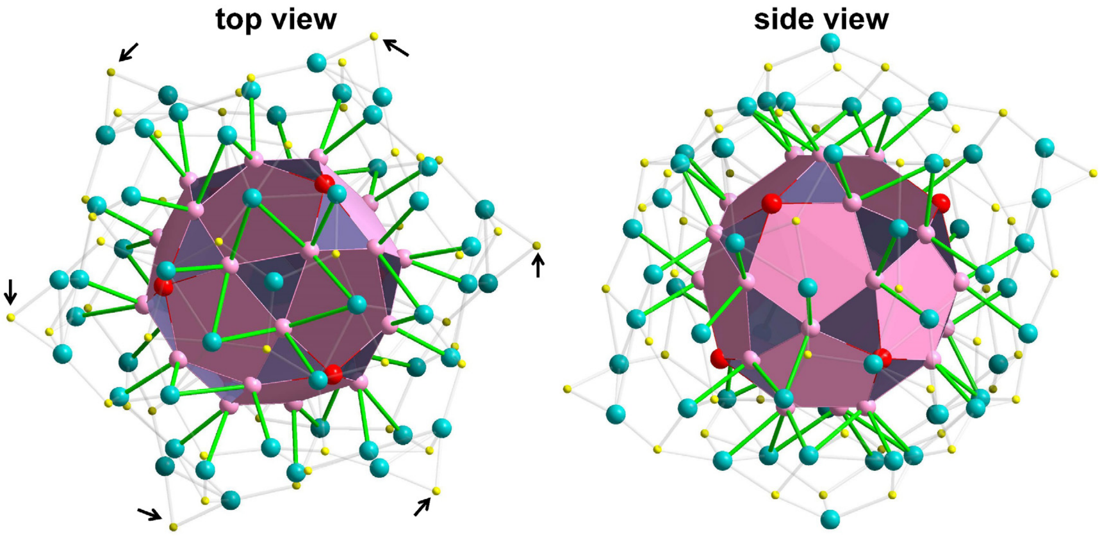

Supplementary Figure 11. Tight coordination (green bonds) of the $(AgCu)_{30}$ shell to the $Ag_{56}S_{48}$

outer shell except for 6 atoms (red). Color codes: green, cyan, pale pink, red, Ag; pale pink, Cu; yellow, S.

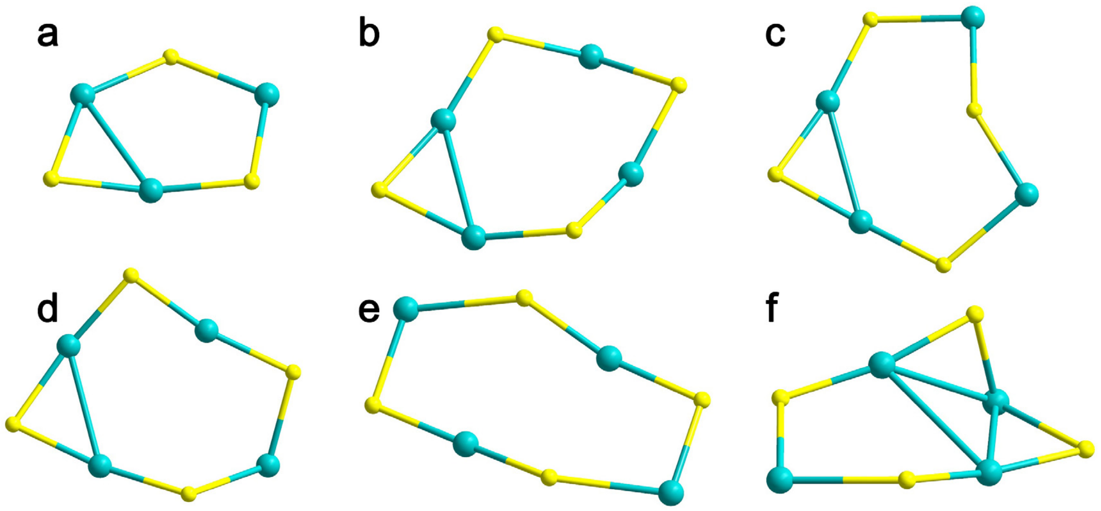

Supplementary Figure 12. Six types of cyclic staples (a ~ f) on the $Ag_{56}S_{48}$ shell. Color codes: cyan, Ag; yellow, S.

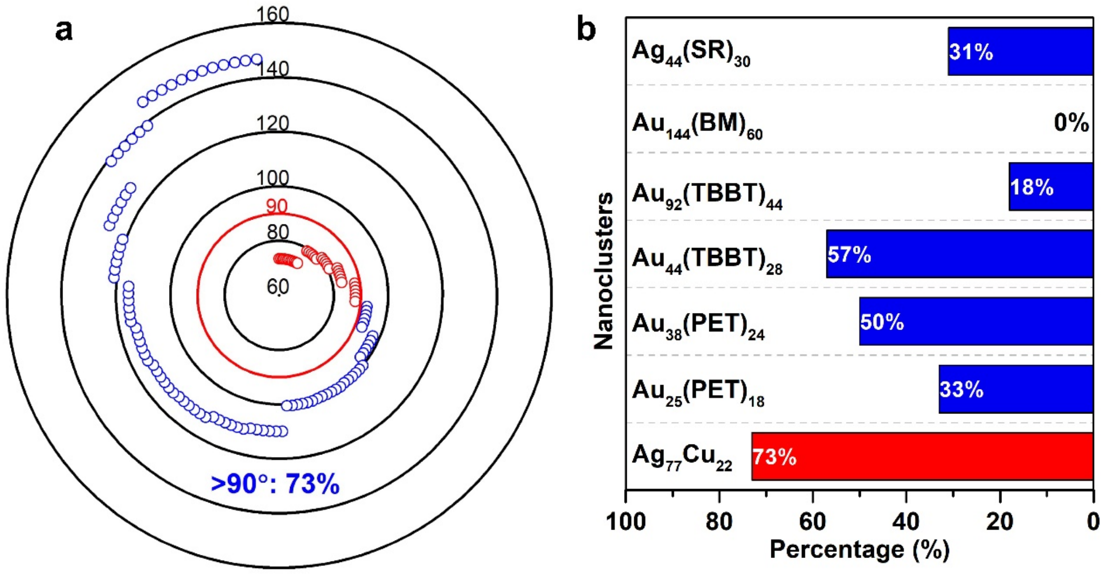

Supplementary Figure 13. (a) The statistics for the Ag-S-Ag angles on the $Ag_{56}S_{48}$ shell. (b) A list showing the percentage of M-S-M angles > 90° for some common NCs.

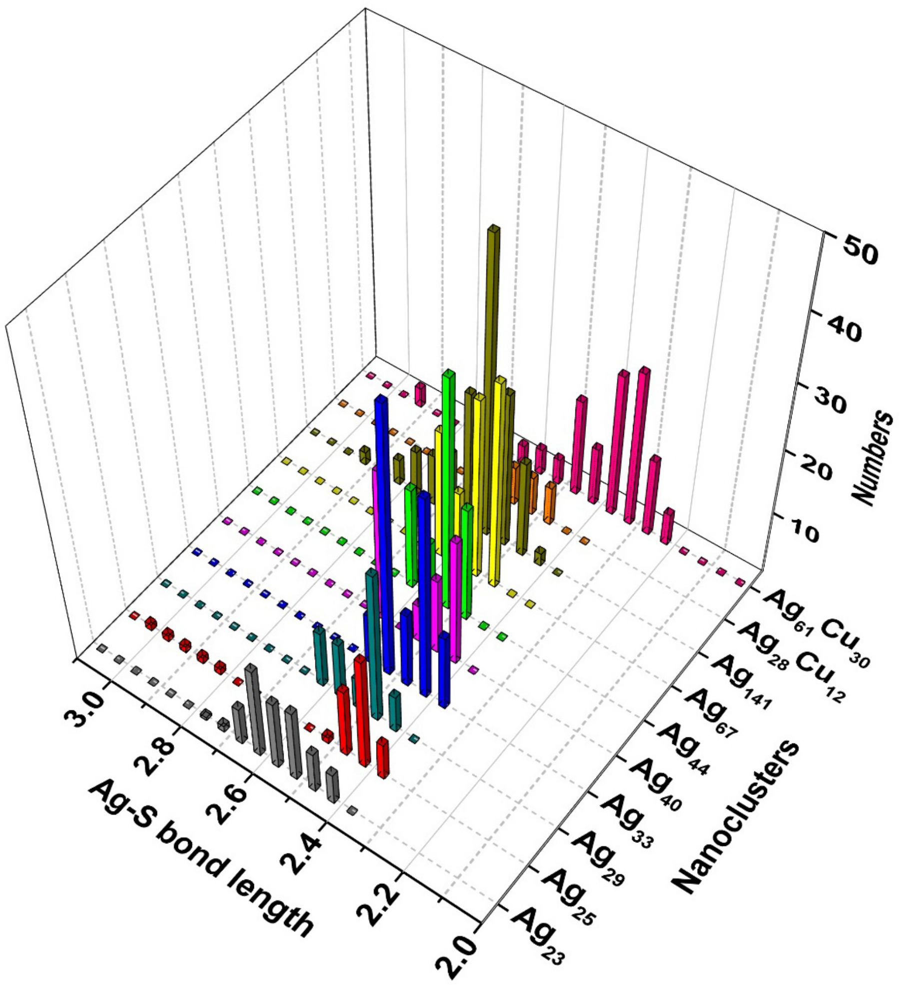

Supplementary Figure 14. Ag-S bonds for some typical Ag or AgCu nanoclusters. The bond length mainly covers the range of 2.3~2.7 Å.

Supplementary Table 1. The H···H interaction distance between interparticle or intraparticle CHT ligands.

	Inter H···H	Intra H···H		Intra H···H
	2.508	2.961	2.490	2.275
	2.468	2.977	2.431	2.631
	2.439	2.865	2.231	2.698
	2.824	2.677	2.784	2.141
Interaction distance (Å)	2.532	2.835	2.366	2.614
	2.532	2.354	2.953	2.789
	2.824	2.856	2.436	
	2.422	2.736	2.820	
	2.468	2.823	2.586	
	2.635	2.603	3.490	
Average (Å)	2.565	2.714		2.525

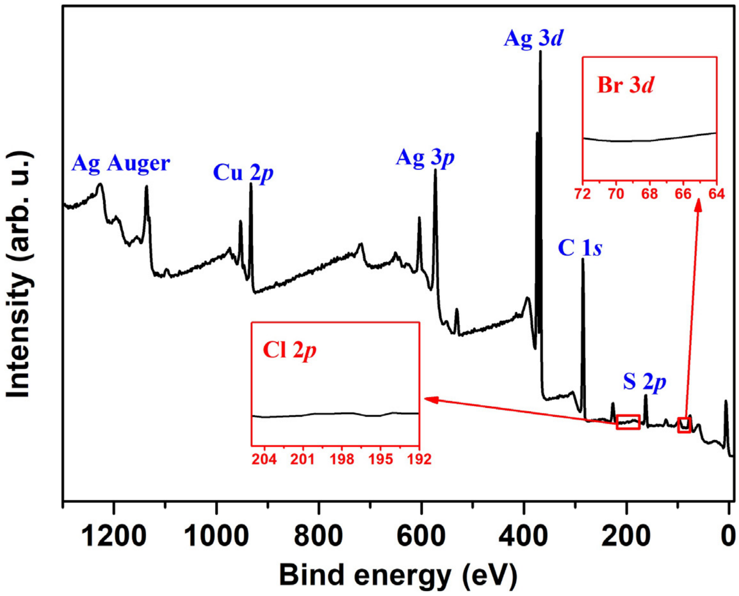

Supplementary Figure 15. XPS patterns of $Ag_{77}Cu_{22}$ crystals.

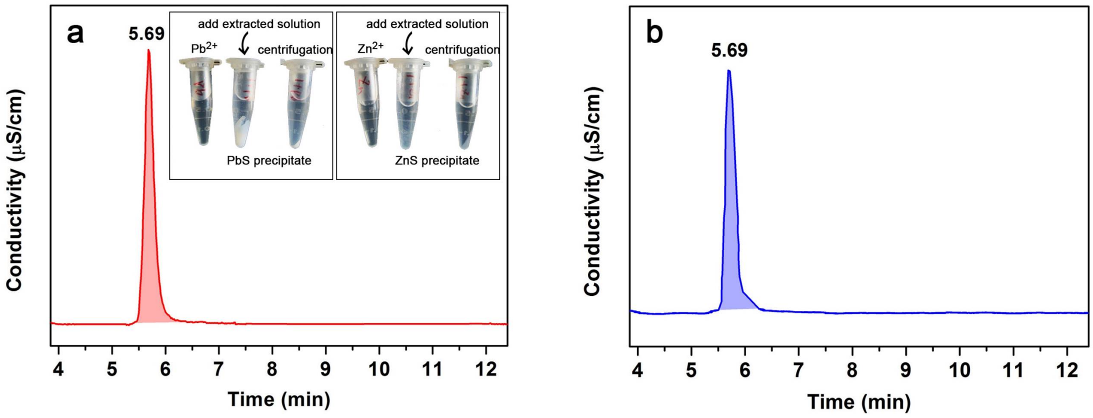

Supplementary Figure 16. Ion chromatograph of the aqueous sample (a) after extracting the reaction mixture and (b) after extracting the solution from the crystals. The peak at 5.69 min (retention time) is attributed to S^{2-} anions. The inset shows the precipitation reaction between the extracted S^{2-} anions and Pb^{2+} or Zn^{2+} ions.

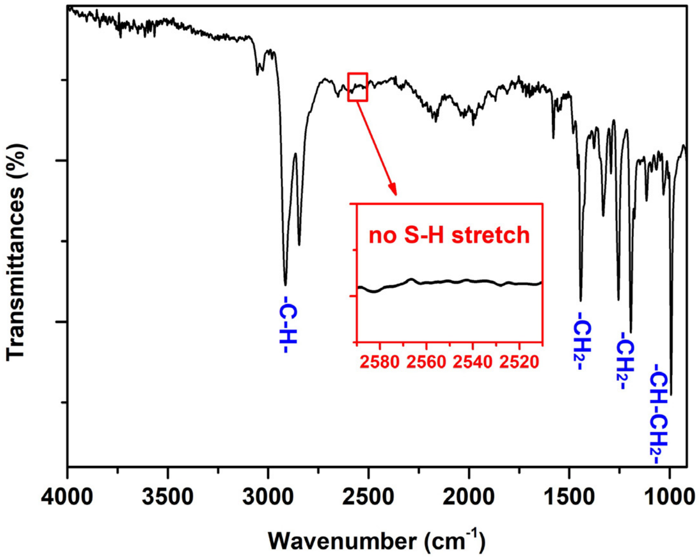

Supplementary Figure 17. Infrared spectrum of $Ag_{77}Cu_{22}$ crystals.

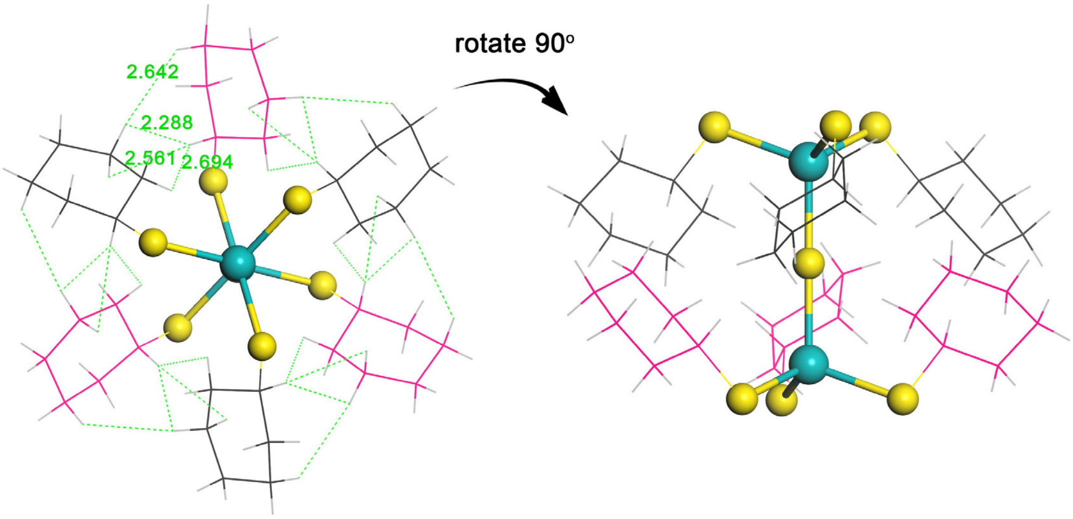

Supplementary Figure 18. The interparticle H···H interactions of the CHT ligands stabilizing the linkage. Right: top view. Left: side view. Color codes: cyan, Ag; yellow, S; gray, C; light gray, H.

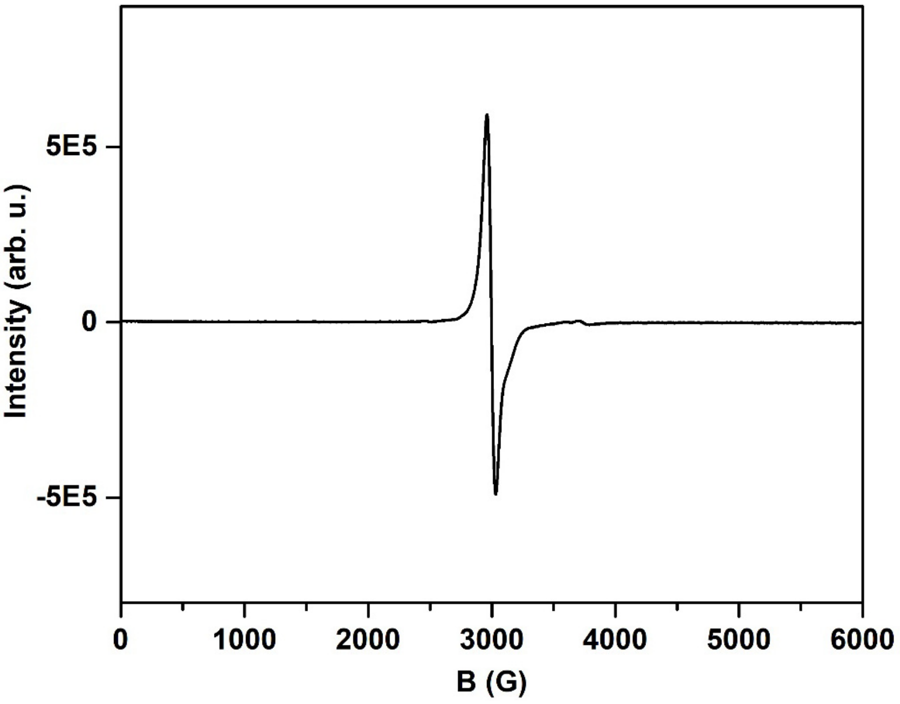

Supplementary Figure 19. EPR spectrum for one single crystal of Au_{25}^{0}.

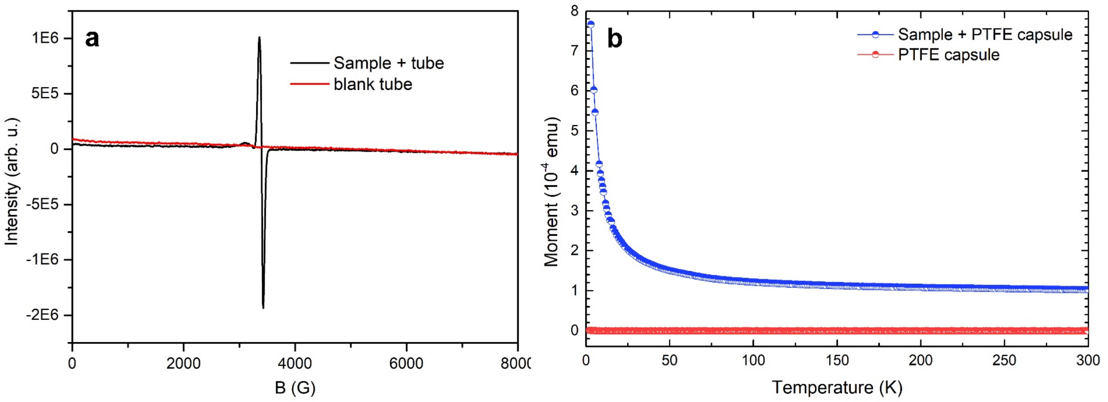

Supplementary Figure 20. (a) The comparison of EPR signals of the sample and the blank tube. (b) The comparison of SQUID curves of the sample and the blank capsule.

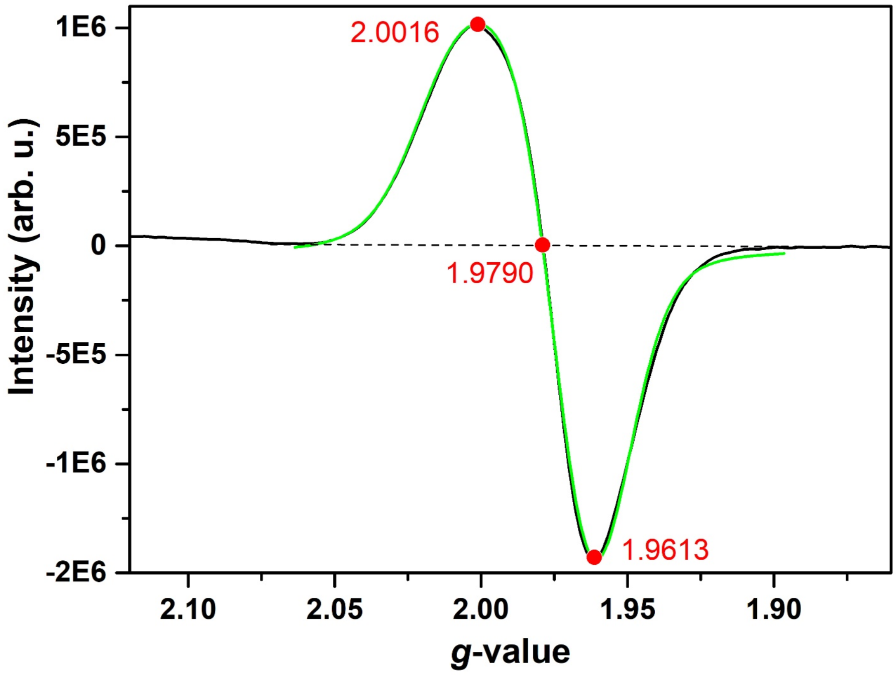

Supplementary Figure 21. Zoomed (black line) and simulated (green line) signals of $Ag_{77}Cu_{22}$. Simulated *g*-values = (2.0072, 1.9671, 1.9659).

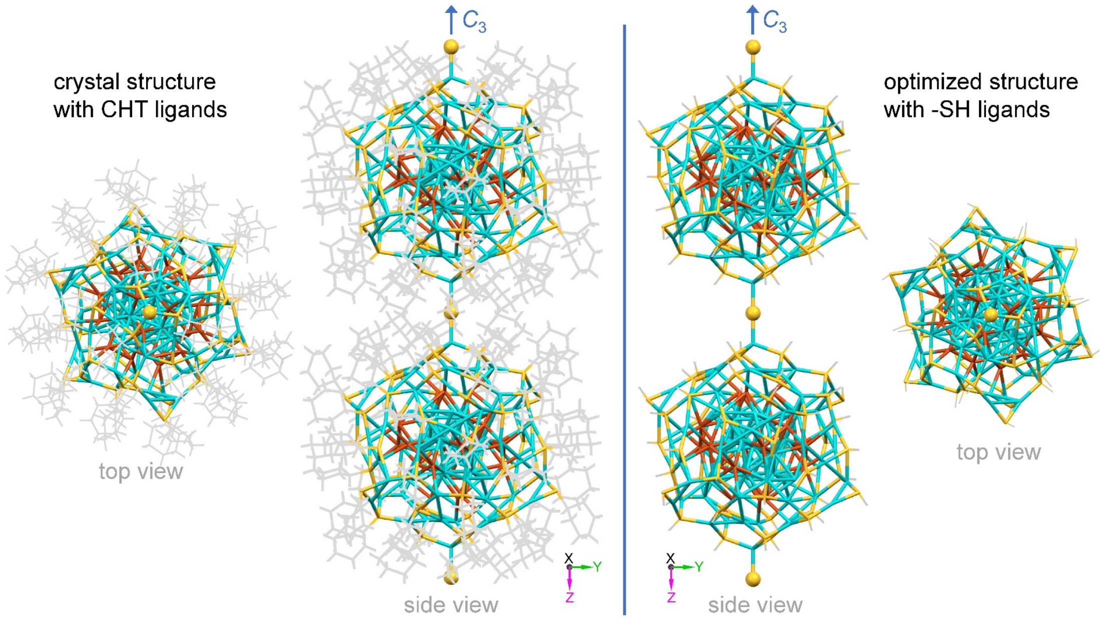

Supplementary Figure 22. Structural comparison of crystal structure and optimized structure of $Ag_{77}Cu_{22}$.

Supplementary Table 2. Values of the main spin density distribution for different atoms in $Ag_{77}Cu_{22}$.

atoms	spin density
linkage S	0.383
Ag atom directly connected with linkage S (Ag1)	0.032
S atom connected with Ag1 (S1)	0.009
Ag atom connected with S1	0.014
kernel metal atoms adjacent to Ag1	0.021
	0.015
	0.014
	0.012

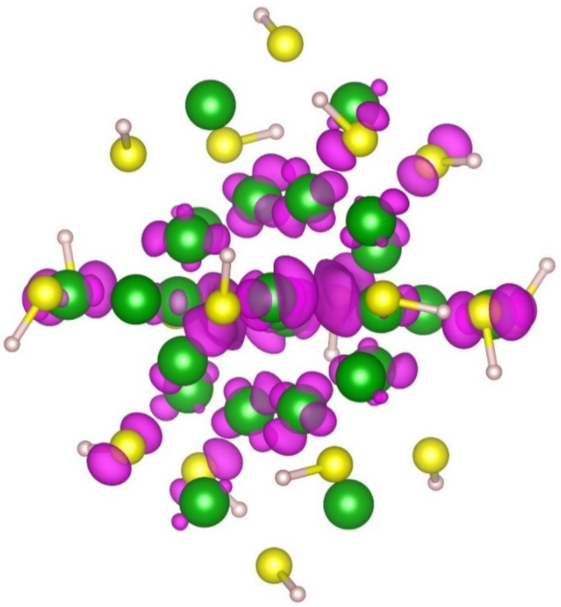

Supplementary Figure 23. The spin density distribution for Au_{25}^0.

Supplementary Table 3. Values of the spin density distribution for different atoms in Au_{25}^0.

Au atom location	spin density
central atom	0.024
icosahedral shell	0.032
	0.030
	0.036
	0.034
	0.039
	0.056
six staple motifs	0.003
	0.019
	0.016
	0.009
	0.012
	0.003

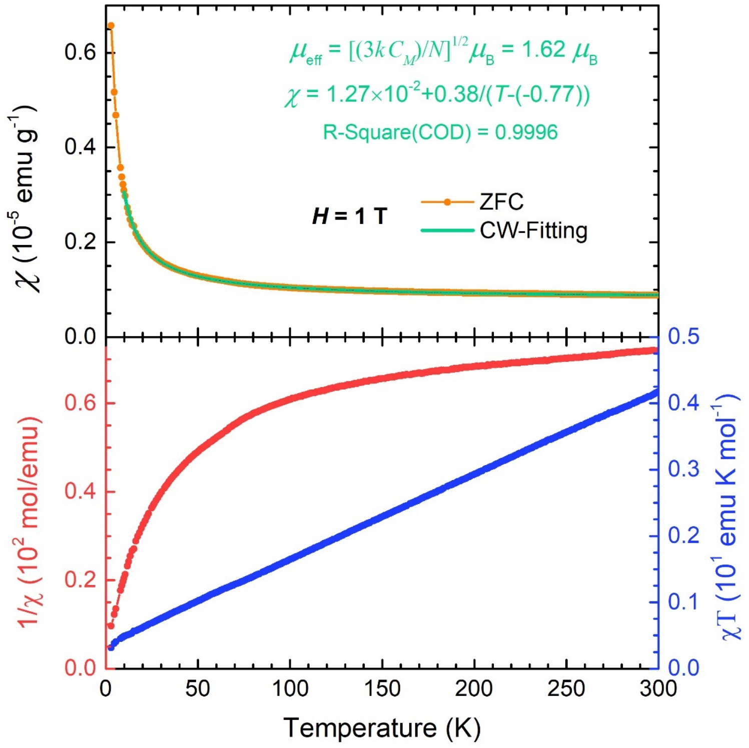

Supplementary Figure 24. Temperature dependence of the magnetic susceptibility of $Ag_{77}Cu_{22}$ crystals.

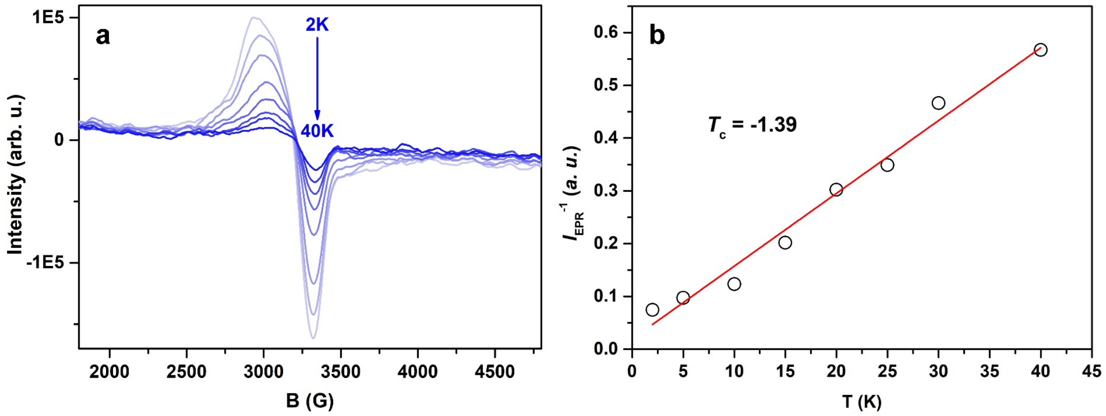

Supplementary Figure 25. (a) Temperature-dependent EPR signals of $Ag_{77}Cu_{22}$ crystals. (b) Plot of I_{EPR}^{-1} –T and the corresponding linear fitting reveal the noninteraction of the spins (T_c = -1.39)[1].

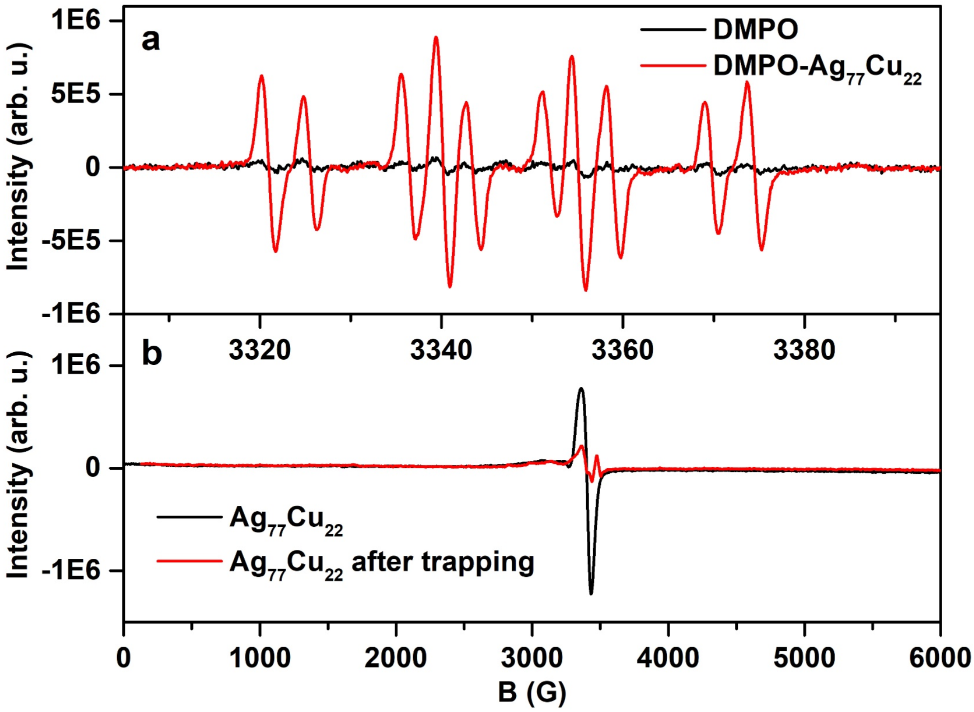

Supplementary Figure 26. (a) EPR spectra measured for an aqueous solution of DMPO treated with $Ag_{77}Cu_{22}$ at room temperature. (b) EPR spectra measured for $Ag_{77}Cu_{22}$ after trapping at 2K.

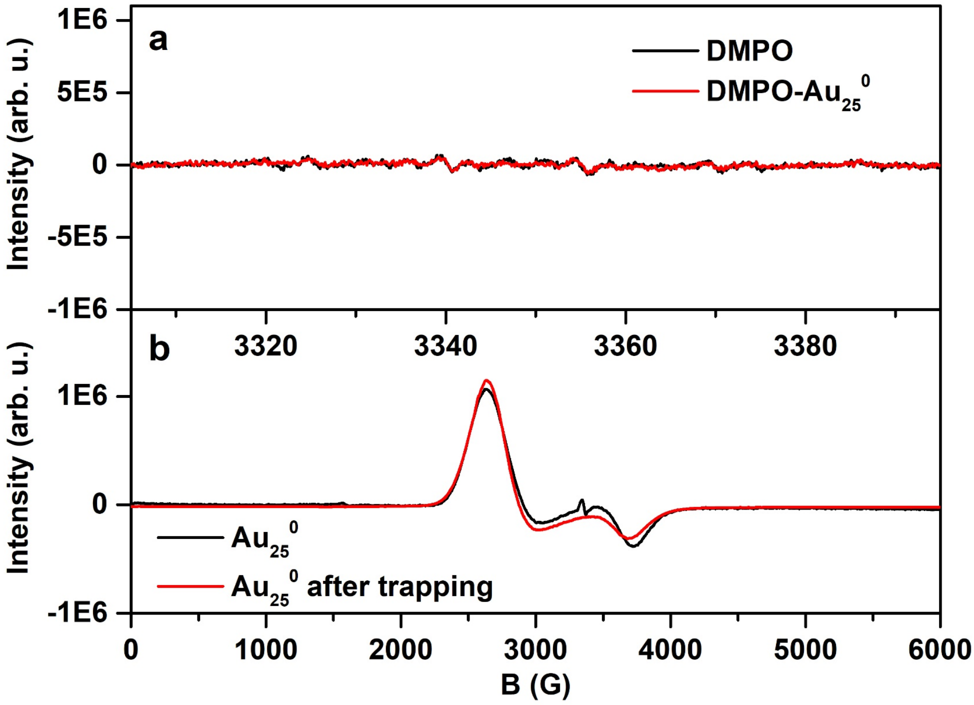

Supplementary Figure 27. (a) EPR spectra measured for an aqueous solution of DMPO treated with Au_{25}^{0} at room temperature. (b) EPR spectra measured for Au_{25}^{0} after trapping at 2K.

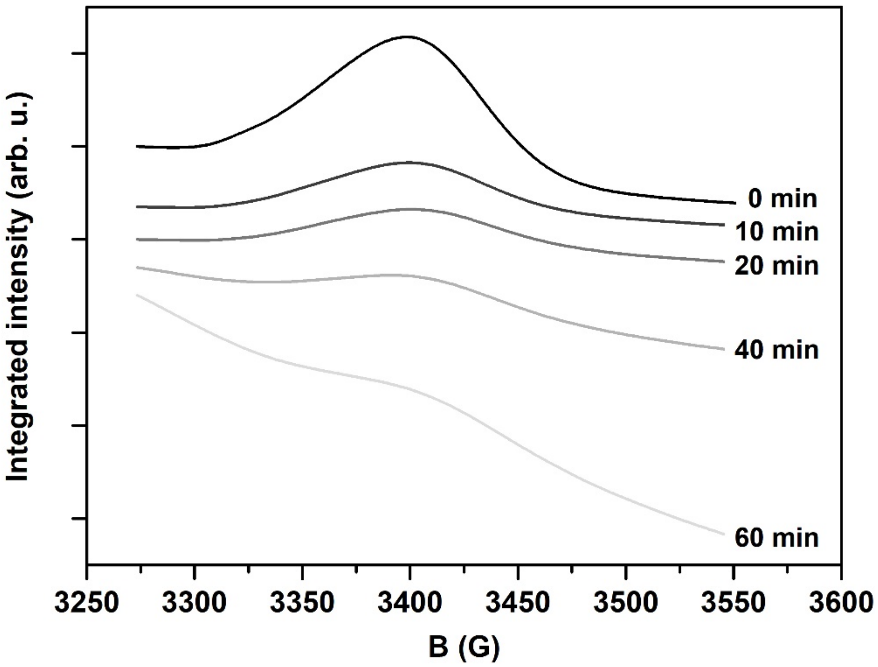

Supplementary Figure 28. Time evolution of the integrated EPR intensity for $Ag_{77}Cu_{22}$ crystals in DCM solution.

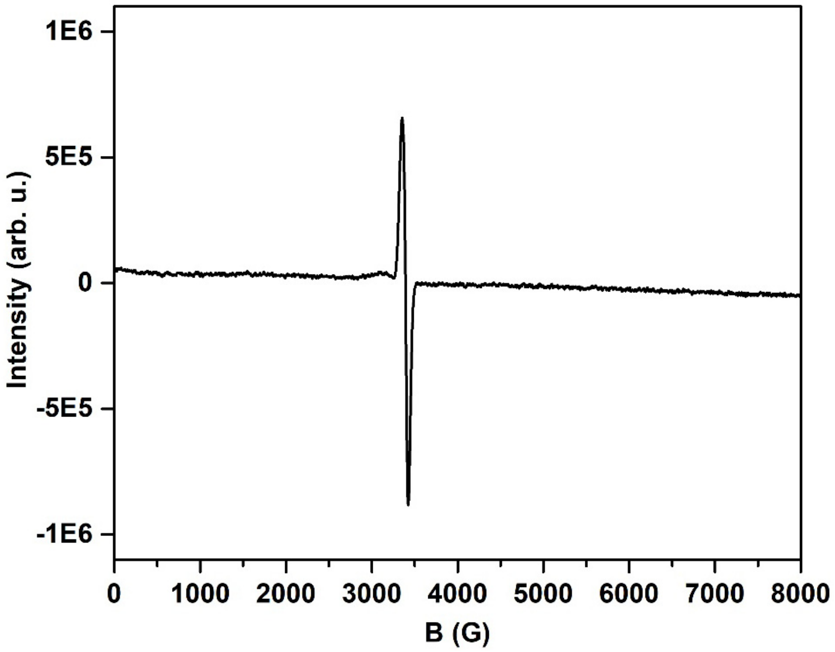

Supplementary Figure 29. EPR spectrum measured for freshly disassembled $Ag_{77}Cu_{22}$ clusters (the signals are majorly assigned to isolated $Ag_{77}Cu_{22}$ clusters since the measurement samples were prepared by dissolving $Ag_{77}Cu_{22}$ crystals in DCM and then promptly removing the solvent).

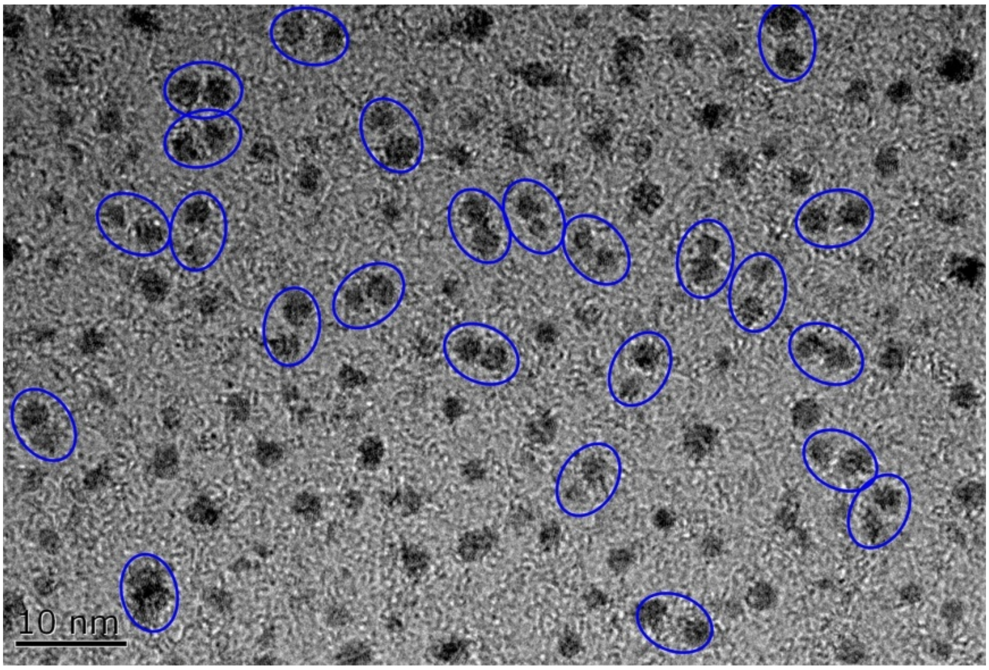

Supplementary Figure 30. TEM image of a fresh solution of $Ag_{77}Cu_{22}$ crystals. Blue circles indicate the pairing of two particles.

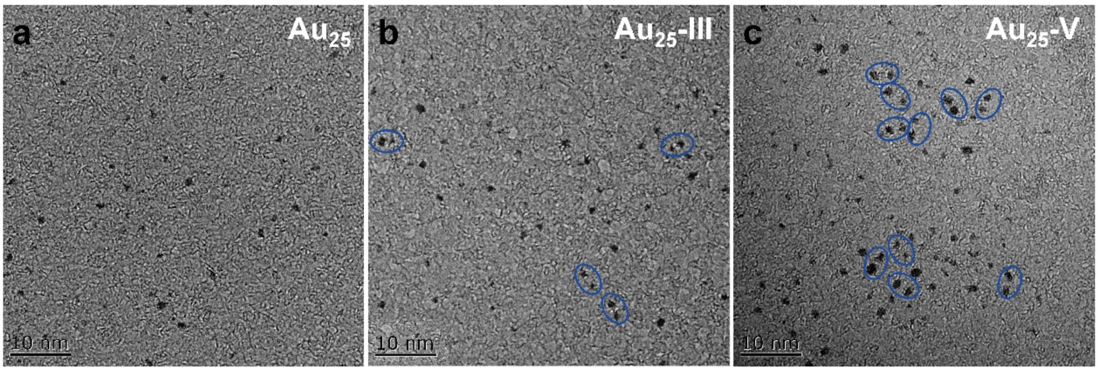

Supplementary Figure 31. TEM images of the solution of (a) Au_{25}, (b) Au_{25}-III, and (c) Au_{25}-V. Blue circles indicate the pairing of two particles.

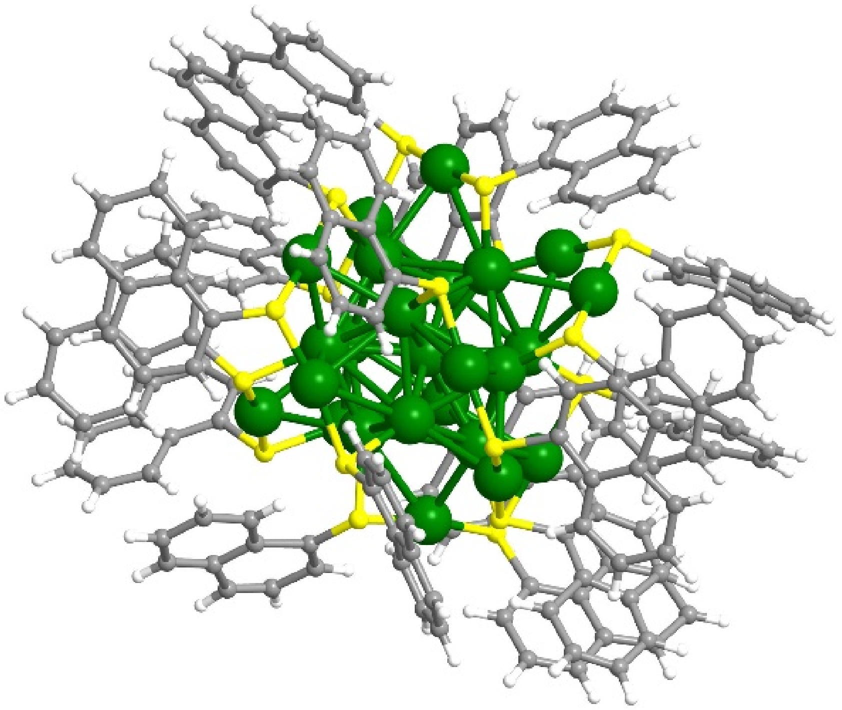

Supplementary Figure 32. Crystal structure of $Au_{25}(Nap)_{18}$. Color codes: Green, Au; yellow, S; gray, C; light gray, H.

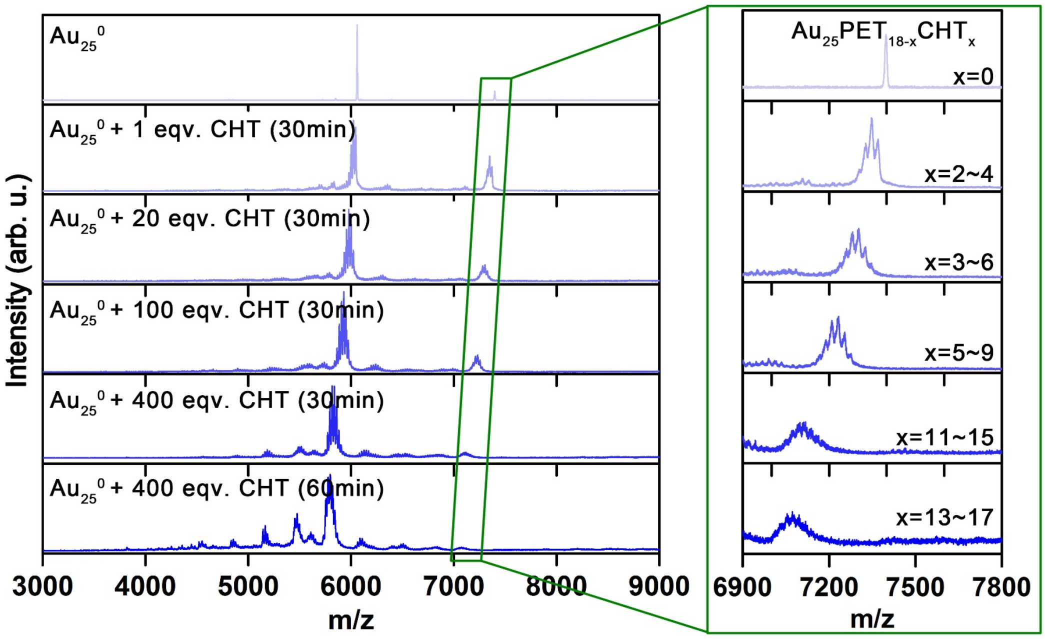

Supplementary Figure 33. MALDI MS spectra for Au_{25}^0 with different CHT substitution numbers.

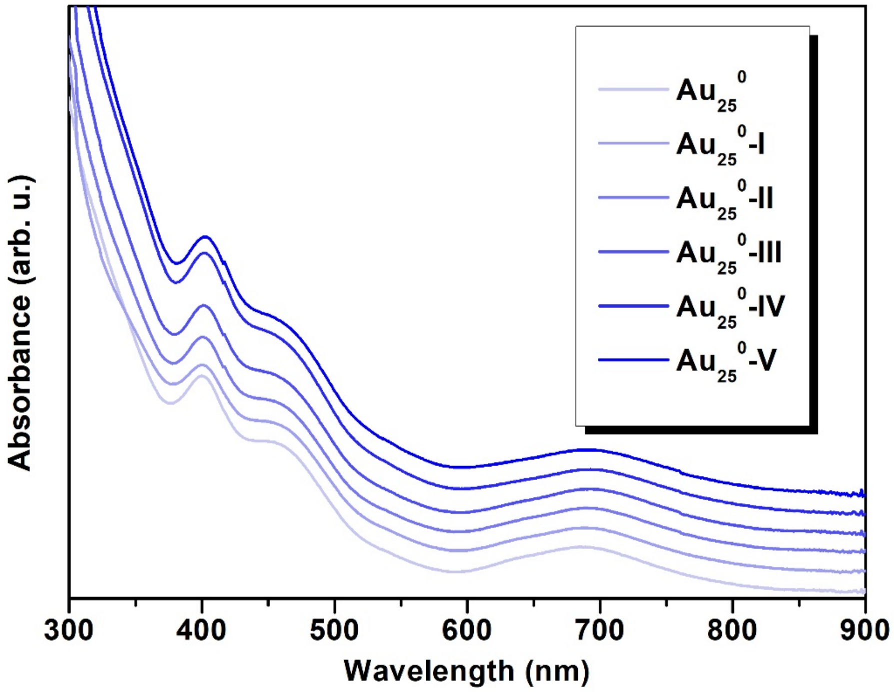

Supplementary Figure 34. UV−Vis spectra for Au_{25}^{0} with different CHT substitution numbers.

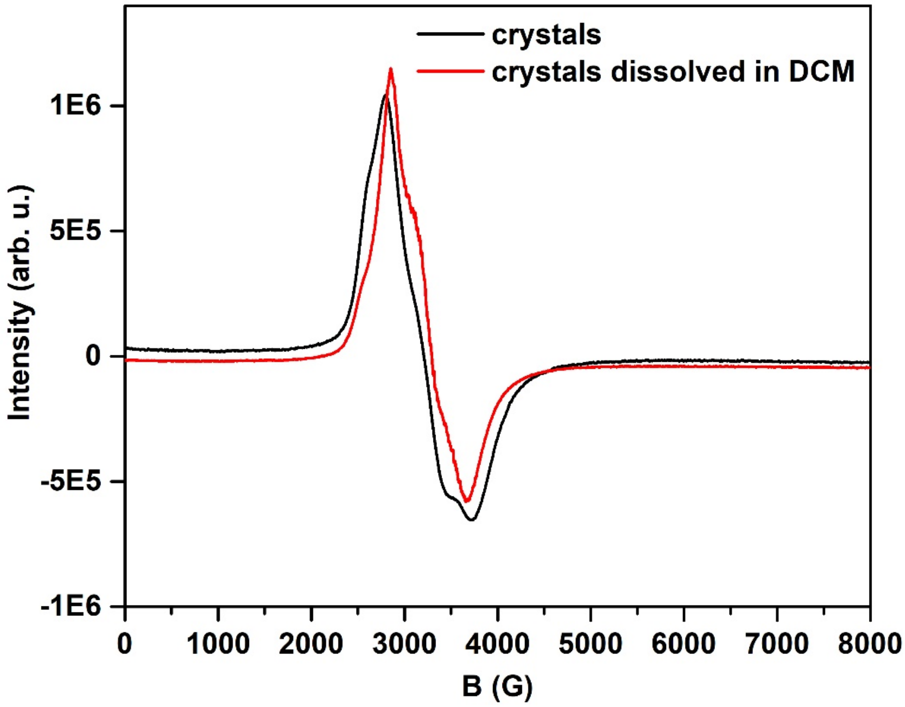

Supplementary Figure 35. EPR spectra measured for $Au_{25}(Nap)_{18}$ in the crystal and solution states.

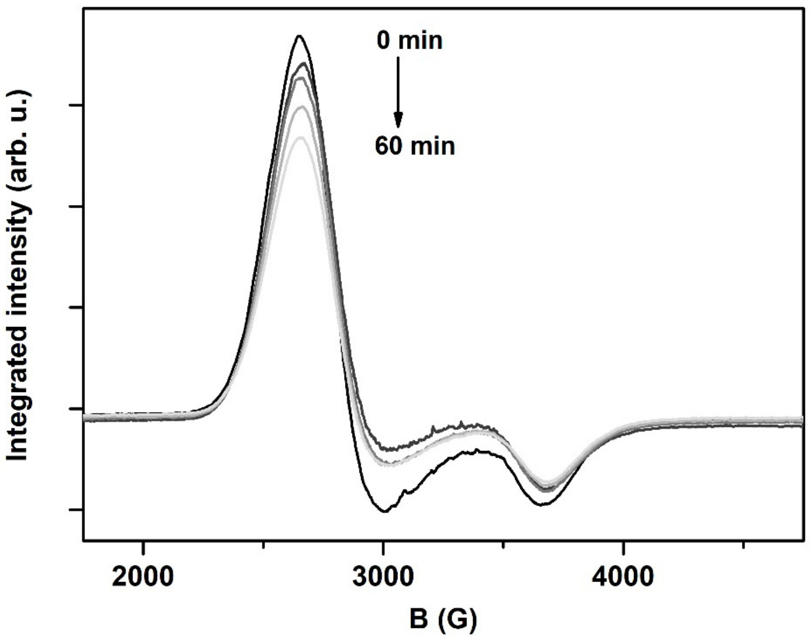

Supplementary Figure 36. Time evolution of the EPR spectra measured for Au_{25}-III in DCM solution.

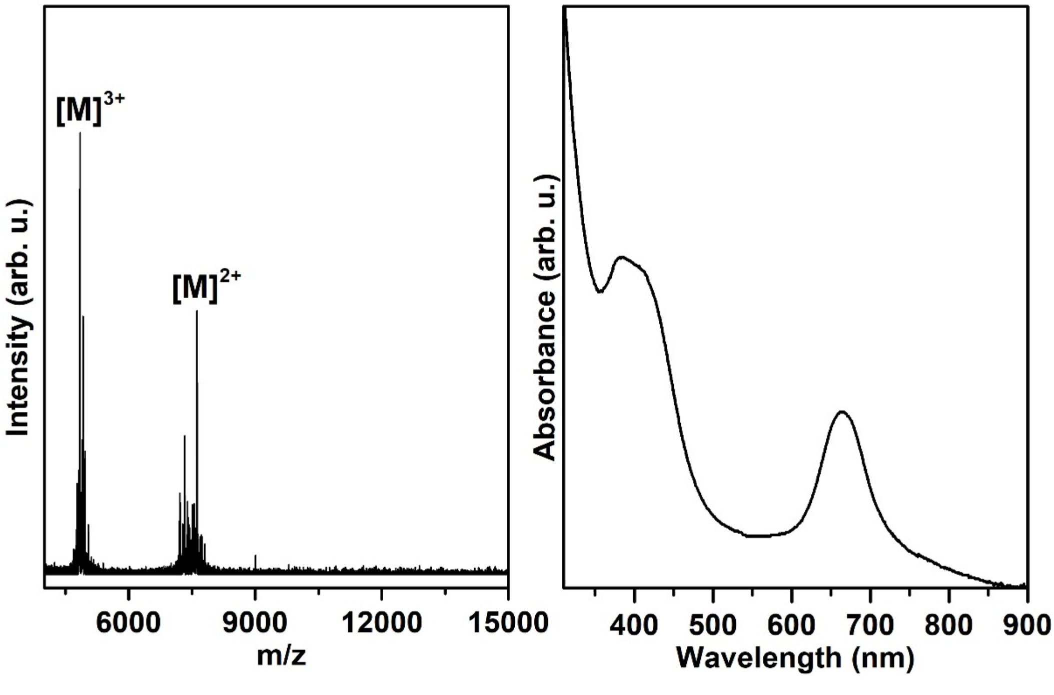

Supplementary Figure 37. ESI-MS and UV-Vis spectrometry for the disassembled $Ag_{77}Cu_{22}$ crystals.

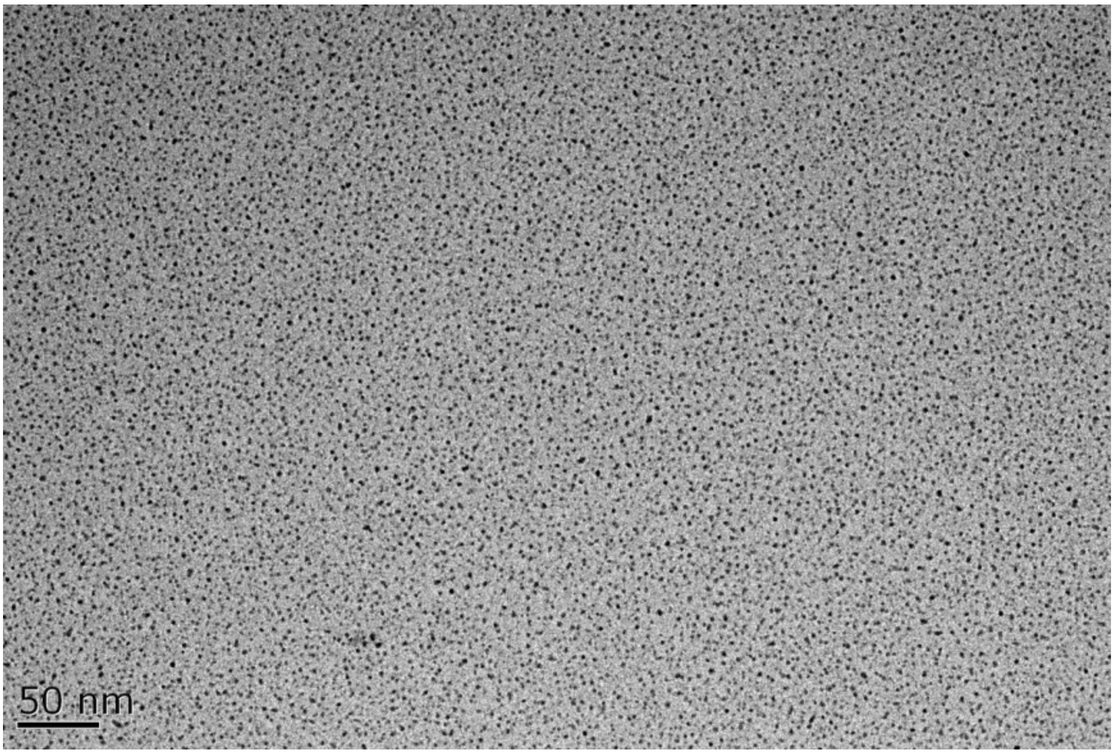

Supplementary Figure 38. Decomposition of the $Ag_{77}Cu_{22}$ crystal chain structure in solution.

Supplementary Table 4. Crystal data and structure refinement for $Ag_{77}Cu_{22}$.

Identification code	191024xn_2_0m	
Empirical formula	C287.99 H527.99 Ag77 Cu22 S49	
Formula weight	15265.58	
Temperature	169.98 K	
Wavelength	1.34139 Å	
Crystal system	Trigonal	
Space group	P-3	
Unit cell dimensions	a = 24.9963(8) Å	α = 90°.
	b = 24.9963(8) Å	β = 90°.
	c = 21.9463(8) Å	γ = 120°.
Volume	11875.3(9) Å^3	
Z	1.00002	
Density (calculated)	2.135 Mg/m^3	
Absorption coefficient	23.282 mm^{-1}	
F(000)	7297	
Crystal size	0.08 x 0.06 x 0.06 mm^3	
Theta range for data collection	3.076 to 55.157°.	
Index ranges	-30<=h<=28, -30<=k<=30, -26<=l<=26	
Reflections collected	119468	
Independent reflections	15122 [R(int) = 0.1647]	
Completeness to theta = 53.594°	99.8 %	
Absorption correction	Semi-empirical from equivalents	
Max. and min. transmission	0.7508 and 0.1171	
Refinement method	Full-matrix least-squares on F^2	
Data / restraints / parameters	15122 / 501 / 658	
Goodness-of-fit on F^2	1.161	
Final R indices [I>2sigma(I)]	R1 = 0.1068, wR2 = 0.2357	
R indices (all data)	R1 = 0.1448, wR2 = 0.2503	

Extinction coefficient	0.000162(14)
Largest diff. peak and hole	6.240 and -3.926 e.Å^{-3}

Supplementary Reference:

1. Agrachev, M. *et al.* Magnetic Ordering in Gold Nanoclusters. *ACS Omega* **2**, 2607–2617 (2017)
